# Evaluation of combination therapy for *Burkholderia cenocepacia* lung infection in different *in vitro* and *in vivo* models

**DOI:** 10.1371/journal.pone.0172723

**Published:** 2017-03-01

**Authors:** Freija Van den Driessche, Bieke Vanhoutte, Gilles Brackman, Aurélie Crabbé, Petra Rigole, Jurgen Vercruysse, Glenn Verstraete, Davie Cappoen, Chris Vervaet, Paul Cos, Tom Coenye

**Affiliations:** 1 Laboratory of Pharmaceutical Microbiology, Ghent University, Ghent, Belgium; 2 Laboratory for Microbiology, Parasitology and Hygiene (LMPH), University of Antwerp, Wilrijk, Belgium; 3 Laboratory of Pharmaceutical Technology, Ghent University, Ghent, Belgium; ENEA Casaccia Research Centre, ITALY

## Abstract

*Burkholderia cenocepacia* is an opportunistic pathogen responsible for life-threatening infections in cystic fibrosis patients. *B*. *cenocepacia* is extremely resistant towards antibiotics and therapy is complicated by its ability to form biofilms. We investigated the efficacy of an alternative antimicrobial strategy for *B*. *cenocepacia* lung infections using *in vitro* and *in vivo* models. A screening of the NIH Clinical Collection 1&2 was performed against *B*. *cenocepacia* biofilms formed in 96-well microtiter plates in the presence of tobramycin to identify repurposing candidates with potentiator activity. The efficacy of selected hits was evaluated in a three-dimensional (3D) organotypic human lung epithelial cell culture model. The *in vivo* effect was evaluated in the invertebrate *Galleria mellonella* and in a murine *B*. *cenocepacia* lung infection model. The screening resulted in 60 hits that potentiated the activity of tobramycin against *B*. *cenocepacia* biofilms, including four imidazoles of which econazole and miconazole were selected for further investigation. However, a potentiator effect was not observed in the 3D organotypic human lung epithelial cell culture model. Combination treatment was also not able to increase survival of infected *G*. *mellonella*. Also in mice, there was no added value for the combination treatment. Although potentiators of tobramycin with activity against biofilms of *B*. *cenocepacia* were identified in a repurposing screen, the *in vitro* activity could not be confirmed nor in a more sophisticated *in vitro* model, neither *in vivo*. This stresses the importance of validating hits resulting from *in vitro* studies in physiologically relevant model systems.

## Introduction

Species belonging to the *Burkholderia cepacia* complex (BCC) are opportunistic pathogens mainly known for causing chronic lung infections in cystic fibrosis (CF) patients and in patients with chronic granulomatous disease [1,2]. *Burkholderia cenocepacia* belongs to the BCC and accounts for approximately 45% of BCC infections in CF patients in the United States [1,2]. The complex interaction between *B*. *cenocepacia* and the CF lung is reflected by the various clinical outcomes and disease severity, from transient colonization to necrotizing pneumonia and sepsis resulting in early death [3]. In addition, respiratory infections caused by *B*. *cenocepacia* are associated with lower survival following lung transplantation [4,5]. Antimicrobial therapy against these infections frequently fails due to *B*. *cenocepacia’s* resistance to many antibiotics and the tolerance typically associated with a biofilm-specific lifestyle [6–8]. Compounds that improve the activity of antibiotics have been called helper compounds [9], potentiators [10], adjuvants [11], or resistance breakers [12]. The aim of the present study was to identify such compounds that reverse tolerance and/or resistance towards tobramycin by increasing the susceptibility of *B*. *cenocepacia* biofilms. To this end, we screened a repurposing library containing compounds that passed human safety assessments and for which the toxicity and metabolic properties are already known [13]. Activity was evaluated in several *in vitro* and *in vivo* assays.

## Material and methods

### Strains and culture conditions

*B*. *cenocepacia* LMG 16656 was maintained on tryptic soy agar plates (TSA, Lab M, Lancashire, UK). Overnight suspensions were obtained by inoculating Mueller Hinton broth (MH, Lab M) with several colonies and incubation with shaking for 18 h. A green fluorescent protein (GFP) producing *B*. *cenocepacia* K56-2 mutant was maintained on Luria-Bertani agar (LB agar, Lab M) supplemented with 800 μg/mL trimethoprim (Ludeco, Brussels, Belgium) [14]. Overnight suspensions were made in LB broth supplemented with 800 μg/mL trimethoprim and inoculated for 23–24 h in a shaker (250 rpm). All cultures were kept aerobically at 37°C.

### Library compounds and antimicrobial agents

The NIH Clinical Collections 1&2 (NIHCC 1&2, Evotec, San Francisco, CA) contain 727 compounds dissolved in 100% dimethyl sulfoxide (DMSO) at a concentration of 10 mM. Miconazole nitrate was obtained from Certa NV (Eigenbrakel, Belgium), meropenem from Astrazeneca (Zoetermeer, The Netherlands), econazole nitrate, ketoconazole, ciprofloxacin, and gentamicin sulfate from Sigma (Bornem, Belgium), oxiconazole nitrate from Santa Cruz Biotechnology (Heidelberg, Germany), and tobramycin from TCI chemicals (Tokyo, Japan). Azoles were dissolved in 100% DMSO (Sigma) and subsequently diluted in MilliQ water (MQ) (Millipore, Billerica, MA) for MIC determination, in physiological saline (PS, 0.9% w/v NaCl) for the checkerboard tests, for the tests with biofilms formed in 96-well microtiter plates (MTPs) and for the *G*. *mellonella* assay, or in GTSF-2 medium for the 3D organotypic human cell culture model [15]. Antibiotic stocks were made in MQ water or PS. All antibiotic solutions were filter sterilized (0.22 μm, Whatman, Dassel, Germany) and immediately used.

### Library screen and identification of hits

*B*. *cenocepacia* LMG 16656 biofilms were formed in round-bottomed 96-well MTPs as described by Van den Driessche et al. [16]; after 24 h of biofilm formation they were treated with one μl library compound (final concentration: 100 μM), 49 μL PS and 50 μL of a 1024 μg/mL tobramycin solution (final concentration: 512 μg/mL). One μL of compound solution was added to 99 μL PS for the experiments carried out in absence of tobramycin. An untreated control, a control for tobramycin treatment alone and a blank were included on every plate. After 24 h, biofilms were rinsed with 100 μL PS and the effect of the treatment was evaluated using CellTiter-Blue staining (CTB, Promega, Leiden, The Netherlands). Fluorescence was measured using a multilabel MTP-reader (Envision, Perkin Elmer LAS, Waltham, MA) as described by Peeters et al. [17]. The blank corrected fluorescence signals generated by *B*. *cenocepacia* biofilms treated with the combination of test compound and tobramycin were compared to the fluorescence signals generated by biofilms that were treated with tobramycin alone. Hits were defined as compounds that caused a decrease in fluorescence signal of ≥ 90% in combination with tobramycin compared to the fluorescence signal generated by biofilms treated with tobramycin alone. Hit compounds were also tested in absence of tobramycin and the fluorescence generated was compared to that from the untreated control.

### Determination of the *in vitro* activity of econazole and miconazole against *B*. *cenocepacia*

To determine the MICs of miconazole and econazole, overnight *B*. *cenocepacia* LMG 16656 cell suspensions were adjusted to OD_595_ of 0.05 and diluted 1:50 in double concentrated MH. Subsequently, 100 μL cell suspension was added to flat-bottomed 96-well MTP (SPL Lifescience, Korea) containing 100 μL of 1/2 serial diluted imidazole solutions (concentration range 0–400 μM). OD_590_ was measured using a multilabel MTP reader (Envision) after 24 h. MICs were determined in triplicate on different days.

Biofilms were formed as described above and treated with 50 μL 200 μM econazole or miconazole (final concentration: 100 μM) and 50 μL 1024 μg/mL tobramycin (final concentration: 512 μg/mL). After 24 h, supernatants were removed and wells were rinsed. Cells were collected from the wells by 2 cycles of shaking (5 min, 700 rpm, Titramax 1000) and sonicating (5 min, Branson 3570E-MT, Branson Ultrasonic Corporation, Banbury, CT). The content of 5 wells was pooled in tubes containing 9 mL PS and the number of CFU per biofilm (CFU/BF) was determined by plating serial 10-fold dilutions on TSA plates. In addition, the potentiating activity of lower concentrations of econazole and miconazole (1 and 10 μM) towards tobramycin, as well as the combination of 100 μM econazole with meropenem (320 and 3200 μg/mL), ciprofloxacin (250 μg/mL) and gentamicin (512 and 2560 μg/mL) was evaluated in the same model system. Experiments were performed in triplicate on different days.

For checkerboard assays biofilms were treated with tobramycin (64–8192 μg/mL) and econazole (25–200 μM) or miconazole (25–200 μM). Potential synergistic activity of these treatments was assessed by calculation of the fractional inhibitory concentration index (FICI) based on metabolic activity determination using CTB. In detail, FICI was calculated as FICI = (C_TOB+IMI_/C_TOB_)+(C_IMI+TOB_/C_IMI_), where C_TOB+IMI_ is the concentration of tobramycin in the presence of the imidazole at which the fluorescence generated is equal to the fluorescence of the blank control, C_TOB_ is the concentration of tobramycin that causes a fluorescence equal to the fluorescence of the blank control, C_IMI+TOB_ is the concentration of the imidazole that causes in the presence of tobramycin a fluorescence signal equal to that of the blank control and C_IMI_ is the concentration of the imidazole that causes a fluorescence signal equal to the fluorescence signal of the blank control. Interactions were scored as synergistic when FICI ≤ 0.5 [18].

### Evaluation of the effect of econazole or miconazole and tobramycin in a 3D organotypic human cell culture model

A 3D human lung epithelial cell culture model was generated by growing A549 cells (ATCC CCL-185) on porous microcarrier beads in a low fluid shear bioreactor, as previously described [19,20]. A549 cells were maintained in GTSF-2 medium supplemented with 2.5 mg/L insulin transferrin selenite (ITS, Sigma-Aldrich), 1.5 g/L sodium bicarbonate, and 10% heat-inactivated fetal bovine serum (FBS, Life Technologies). All cultures were grown at 37°C under 5% CO_2_ conditions. Infection studies were performed using 3D cultures grown for 11 to 14 days, and at the time of infection cell culture medium without FBS and supplemented with 0.2% rhamnose was used. 3D cell cultures containing 2.5 x 10^5^ cells in 250 μL per well were transferred to 48-well plates (SPL Life Sciences). An overnight culture of a GFP-producing *B*. *cenocepacia* K56-2 mutant [14] was resuspended in cell culture medium and added to the 3D cells at a multiplicity of infection of ~15:1. Treatments (156 μg/mL tobramycin and/or 10 μM econazole or miconazole) were added to the cell culture medium at the start of the infection. Plates were incubated at 37°C under 5% CO_2_ conditions for 1 h, rinsed with Hanks’ Balanced Salt Solution (HBSS, Life Technologies) and subsequently fresh cell culture medium containing the treatment reagents was supplied. After 16 h additional incubation, the number of *B*. *cenocepacia* that associated with the 3D cells was determined. To this end, the content of each well was transferred to new wells to avoid inclusion of bacteria attached to the plastic surface. Cultures were rinsed twice with HBSS followed by the addition of 0.1% Triton X-100, vigorous mixing and plating. The experiments were performed in the absence and presence of 3D cells, under otherwise identical conditions, to assess the effect of tobramycin and/or imidazoles on the inhibition of bacterial association with an abiotic or biotic surface, respectively. Controls with no antimicrobials and uninfected 3D cell cultures were included in every assay. Assessment of the overall 3D cell culture model integrity and visualization of GFP-producing bacteria was performed using an EVOS FL Auto Imaging System (Life Technologies) at a magnification of 300x. In addition, the 3D lung epithelial cell viability was evaluated based on a lactate dehydrogenase (LDH) assay. The LDH activity assay kit (Sigma) was used to measure the release of cytosolic LDH by 3D lung epithelial cells following exposure to *B*. *cenocepacia* K56-2. Medium from 3D aggregates infected with *B*. *cenocepacia* for 17 h was centrifuged for 15 minutes at 3700 rpm, where after the supernatants was used for LDH quantification following the manufacturer’s instructions. A standard curve using NADH was included. As a positive control, 2.5 x 10^5^ 3D lung epithelial cells were lysed with 0.1% Triton-X100 in 250 μL (the same volume as for the test conditions). The experiments were performed in triplicate on different days. The data are presented as a percentage of LDH release from the positive control.

### Evaluation of the potentiating effect in a *Galleria mellonella* infection assay

*In vivo* activity of tobramycin and econazole or miconazole was evaluated in the *G*. *mellonella* infection assay, as described by Brackman et al [21]. *G*. *mellonella* larvae (Hengelsport de Poorter, Ghent, Belgium) were kept at 4°C in the dark on wooden flakes prior to use and divided in groups with similar weight distribution (300–450 mg, 450–550 mg or 550–650 mg). Equal amounts of larvae were taken from each group to compose new groups containing at least 12 larvae for the infected groups and 6 for the uninfected groups. Overnight cultures of *B*. *cenocepacia* LMG 16656 were centrifuged, resuspended in PS and adjusted to 10^7^ CFU/mL. Larvae were injected with 10 μL cell suspension in the last left proleg and 10 μL of the treatment solution (512 μg/mL tobramycin, 50 μM econazole, 50 μM miconazole, or combinations) in the last right proleg using a syringe (BD ultrafine insulin syringes, Becton Dickinson, Erembodegem, Belgium). Larvae were incubated at 37°C and survival was scored after 24, 48 and 72 h. Larvae were considered dead when no movement was observed in response to touch. To determine whether the treatment caused a reduction in the number of CFU/larvae 24 h post infection (*p*.*i*.*)*, larvae were homogenized (Polytron, Kinematica, Eschbach, Germany), serially diluted and plated on *Burkholderia cepacia* selective agar (Thermo Scientific, Erembodegem, Belgium).

### Development of a formulation for inhalation containing tobramycin and miconazole

In contrast to tobramycin which is highly soluble in water, miconazole is very slightly soluble in water [22]. Therefore, it was decided to formulate the combination tobramycin-miconazole as a suspension for nebulization. In order to decrease the particle size of the suspended miconazole particles (i.e., down to desired particle size for deposit in the lungs), the wet bead milling technique was applied. The formulation was prepared by weighing 330 mg tobramycin and 15.4 mg or 61.6 mg miconazole into a 20 mL vial, followed by the addition of 1 mL Tween 80 solution (1 mg/mL) (Fagron, Waregem, Belgium), 4 mL PS, and 10 g zirconium oxide beads (diameter 0.5 mm, Netzsch Feinmahltechnik, Selb, Germany). Subsequently, the vials were placed on a roller-mill (Peira, Beerse, Belgium) and grinding was performed at 150 rpm for 72 h. After milling, the microsuspension was separated from the grinding pearls by pipetting. The particle size distribution of miconazole was measured in triplicate by laser diffraction (Mastersizer S long bench, Malvern Instruments, Worcestershire, UK). The wet dispersion technique was applied using the 300RF lens (Malvern Instruments, Worcestershire, UK). The powders were dispersed in a solution of 0.2% Tween 80 in Miglyol 812 and subsequently vortexed and sonicated in order to eliminate agglomerates. In addition, biofilms formed in 96-well MTPs were treated with the formulation in a dilution of 1 in 129 (corresponding to 512 μg/mL tobramycin and 200 μM miconazole) to confirm the antibacterial activity. Experiments were performed six times independently and the effect was quantified by plate counts.

### Mouse lung infection model

Animals were treated in accordance to the guidelines provided by the University of Antwerp and the European Directive for Laboratory Animal Care (Directive 2010/63/EU of the European Parliament). The laboratory Animal Ethics Committee of the University of Antwerp authorized and approved all animal experimentation in this study (file 2014–81). Female inbred BALB/c ByJRj mice of 8–9 weeks old (Janvier Labs, Le Genest-Saint-Isle, France) were kept under standard pathogen-free conditions with a constant temperature of 20–25°C, an average humidity of 50–60%, a 12 h dark-light cycle and food and drinking water ad libitum. Mice were rendered transiently leukopenic with cyclophosphamide (Endoxan, Baxter) at 150 mg/kg body weight (bw) given intraperitoneally 4 days prior to infection and 50 mg/kg bw administered both 2 days prior to infection and on the day of infection. To prepare the inoculum for intratracheal infection, a bacterial cryostock was thawed, diluted in LB broth (10^5^ CFU/mL) and incubated for 24 h in a shaken incubator (25 rpm) at 37°C in a 14 mL vent cap sterile tube. Cells were harvested by centrifugation after 24 h and diluted in PBS to 2 x 10^7^ CFU/mL. Before intratracheal challenge with *B*. *cenocepacia*, mice were anesthetized with isoflurane (Halocarbon, Norcross, GA) and all efforts were made to minimize suffering. Anesthetized mice were inoculated intratracheally with *B*. *cenocepacia* (10^6^ CFU/mouse) in 50 μL of PBS according to Dupage et al. [23],. Thereafter, infected mice were treated intranasally with 40 μl of the formulations described above, i.e. vehicle (0.02% Tween 80 dissolved in PS), tobramycin (66 mg/mL, corresponding to 120 mg/kg bw), miconazole (3.08 mg or 12.32 mg/mL corresponding to 5.6 and 22.4 mg/kg bw miconazole, respectively), and the combination, at 1 h, 24 h and 48 h *p*.*i*. according to Southam et al. [24]. Mice were observed daily for functional behavior (i.e. fur quality, posture, state of activity and bw) and pneumonia symptoms (i.e. respiratory frequency). *B*. *cenocepacia* infected mice were sacrificed at day 3 *p*.*i*. by cervical dislocation. Lung, spleen and liver were excised, weighed and homogenized in 5 mL PBS and were used for the enumeration of CFU on LB agar or *Burkholderia cepacia* selective agar.

### Statistics

Data are expressed as the mean ± standard error of the mean (SEM). SPSS version 23 software was used and data were analyzed using the Kruskal-Wallis test. P-values < 0.05 were considered statistically significant.

## Results

### Library screen against *B*. *cenocepacia* biofilms in combination with tobramycin

Screening the NIHCC 1&2 against *B*. *cenocepacia* biofilms in the presence of tobramycin allowed us to identify 60 hits (8.2%). In the presence of tobramycin these hit compounds caused a decrease of ≥ 90% in fluorescence signal after CTB staining compared to treatment with tobramycin alone (S1 Table). Hits were classified in four groups according to their therapeutic indication: anti-infective agents (n = 11), antipsychotics and antidepressants (n = 23), antineoplastic and/or hormonal drugs (n = 6), and a miscellaneous group (n = 20) (S2 Table). Subsequently, *B*. *cenocepacia* biofilms were treated with the hit compounds alone; 19 compounds did not cause a significant decrease in fluorescence, indicating they are true potentiators. In contrast, 41 compounds caused a significant reduction in fluorescence compared to the untreated control suggesting their potentiating activity is at least partially due to an intrinsic activity against *B*. *cenocepacia* biofilms (S2 Table).

### Antifungal imidazoles are potentiators *in vitro*

Four antifungal imidazoles, i.e. econazole, miconazole, oxiconazole, and ketoconazole were identified as hits in the screening. Treatment of biofilms with econazole alone reduced the fluorescence significantly compared to the untreated control. Miconazole, oxiconazole, and ketoconazole did not cause a significant reduction in fluorescence compared to untreated control (S2 Table).

**Fig 1:**
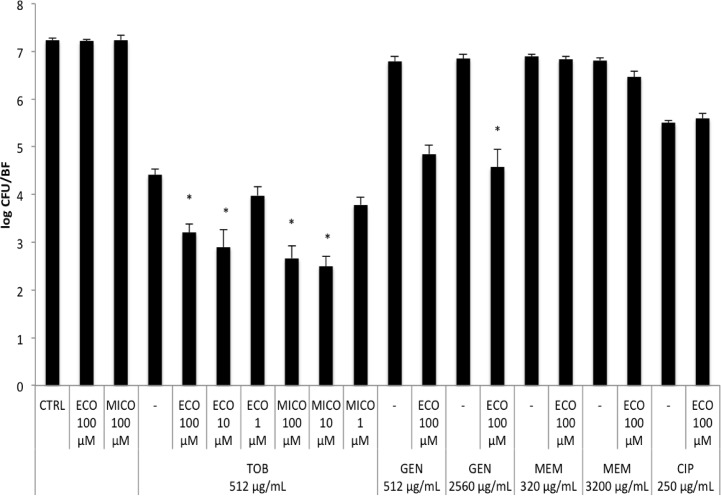
Treatment of mature biofilms formed in 96-well MTPs. Number of CFU per biofilm after treating mature biofilms of *B*. *cenocepacia* LMG 16656 for 24 h with 100 μL PS (untreated control, CTRL), 1, 10, 100 μM econazole (ECO) or 1, 10, 100 μM miconazole (MICO), in combination with 512 μg/mL tobramycin (TOB), 512 and 2560 μg/mL gentamicin (GEN), 320 and 3200 μg/mL meropenem (MEM), or 250 μg/mL ciprofloxacin (CIP). Asterisks indicate a significantly different number in log CFU/BF compared to treatment with the respective antibiotic alone (P value < 0.05). (Data shown are average; n ≥ 3; error bars indicate SEM).

Subsequently, *B*. *cenocepacia* biofilms were treated with 100 μM econazole or miconazole in combination with 512 μg/mL tobramycin and the antimicrobial effect was quantified by plate counts. Treatment with econazole or miconazole alone did not cause a reduction in the number of CFU/BF. However, in combination with tobramycin, a significant reduction in the number of CFU/BF was observed, compared to treatment with tobramycin alone (Fig 1). Lower concentrations of econazole and miconazole were tested for potentiating activity towards tobramycin; there was no potentiator activity for 1 μM, while 10 μM econazole or miconazole were equally effective as 100 μM (Fig 1). The potentiator activity of econazole was subsequently investigated in combination with other antibiotics; 100 μM econazole caused a statistically significant reduction in CFU/BF in combination with 2560 μg/mL gentamicin, but not with 512 μg/mL gentamicin. Econazole did not potentiate the activity of meropenem or ciprofloxacin (Fig 1). In addition, we confirmed that treatment with econazole or the combination tobramycin–econazole did not lead to induced dispersion (S1 Fig).

The MIC for econazole and miconazole against *B*. *cenocepacia* exceeded 200 μM. Subsequently, checkerboard assays were performed against biofilms of *B*. *cenocepacia* and showed that in the presence of the imidazoles, the concentration of tobramycin necessary to completely eradicate biofilms decreased substantially. As the imidazoles alone did not eradicate biofilms in the concentrations tested, and as higher concentrations could not be tested due to solubility issues, exact FICIs could not be calculated.

### Evaluation of the activity of the combination treatment in a 3D organotypic cell culture model

**Fig 2:**
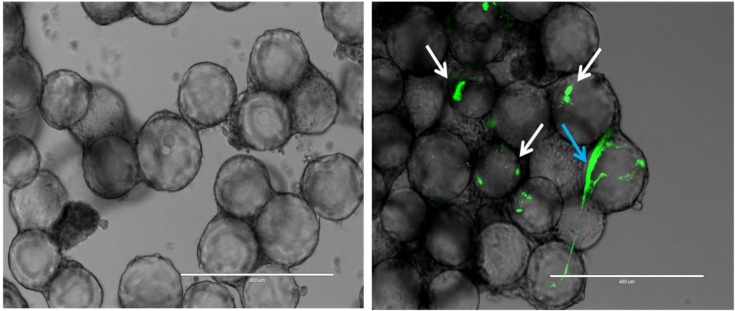
Infection of 3D lung epithelial cells. Left image showing an uninfected control where the microcarrier bead scaffolds are covered with A549 human lung epithelial cells, right image shows the 3D lung epithelial cells 17 h *p*.*i*., green dots (white arrows) indicate intracellular growth of *B*. *cenocepacia* K56-2, while the large green structure (blue arrow) suggests that biofilm-like structures are formed. Magnification is 300x. Scale bar is 400 μm.

*B*. *cenocepacia* K56-2 infected 3D lung epithelial cells without significantly affecting the 3D model integrity (Fig 2) and host cell viability (S2 Fig) untill 17 h post infection. Specifically, based on the LDH release assay less than 10% cell death of infected cultures was observed, which was similar to that observed in the non-infected controls (S2 Fig).

*B*. *cenocepacia* associated with the 3D lung epithelial cells both as dense clusters associated with single host cells and as structures that were spread out over multiple epithelial cells (Fig 2).

**Fig 3:**
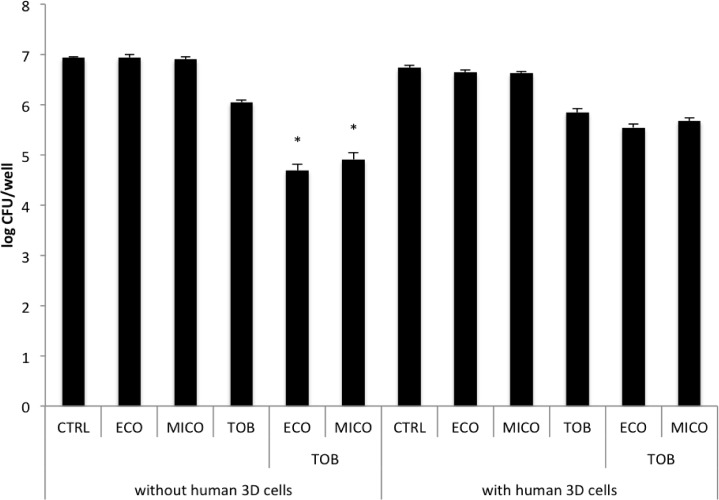
Effect of treatment in the 3D model. Effect of treatment with 10 μM econazole (ECO), 10 μM miconazole (MICO), 156 μg/mL tobramycin (TOB) or the combination on inhibition of biofilm formation of *B*. *cenocepacia* K56-2 with a plastic surface (left, number of biofilm cells per well) or the effect on the treatment on the number of bacterial cells associated with human 3D lung epithelial cells (right, number of host associated cells per well). Asterisks indicate a significantly different number in CFU/well compared to treatment with tobramycin (P value < 0.05). (Data shown are average; n ≥ 3; error bars indicate SEM).

3D lung epithelial cells were exposed to 10, 50 and 100 μM of the imidazoles in the absence of *B*. *cenocepacia* for 17 h (S3 Fig). The epithelial cells completely detached from their microcarrier bead scaffolds when exposed to 50 or 100 μM econazole or miconazole; this was not observed when cells were exposed to 10 μM of the imidazoles (S3 Fig) and the latter concentration was chosen for further experiments. High concentrations of tobramycin (1000 μg/mL) did not affect the 3D lung model integrity (S3 Fig). Adding 10 μM econazole or miconazole led to an additional reduction in *B*. *cenocepacia* biofilm formation in MTP in combination with 156 μg/mL tobramycin (test concentration of tobramycin determined in preliminary experiments, S4 Fig), and this concentration was used to assess the number of viable and culturable bacteria associated with 3D cells. When the combination treatment was tested in this model, no potentiating effect of miconazole and econazole was observed, while the activity of tobramycin was similar as in the MTP (Fig 3).

### *G*. *mellonella* survival assay

**Fig 4:**
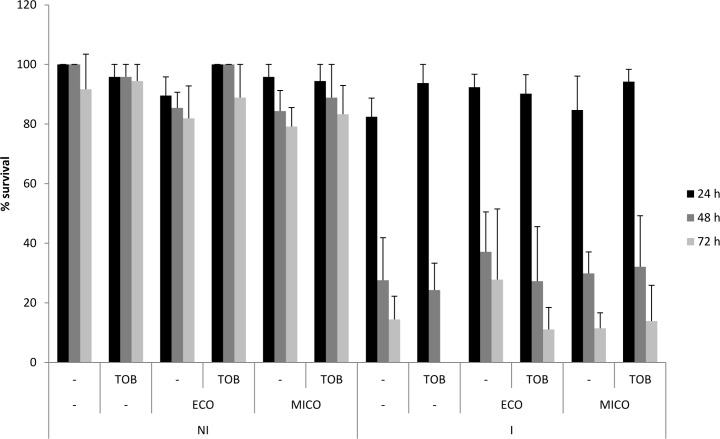
*G*. *mellonella* survival assay. Percentage survival of *G*. *mellonella* in uninfected control groups (NI) and infected groups (I) 24, 48, and 72 h after administration of the different treatments (tobramycin (TOB, 512 μg/ml), econazole (ECO, 50 μM), miconazole (MICO, 50 μM) or the combinations) and/or infection. (Data shown are average, n = 6, error bars indicate SEM).

*G*. *mellonella* larvae were injected with *B*. *cenocepacia* and treated with 10 μL vehicle (PS), 512 μg/mL tobramycin, 50 μM miconazole, 50 μM econazole, tobramycin combined with miconazole, or tobramycin combined with econazole. No significant decrease in survival after 24, 48, and 72 h was observed in the non-infected groups that received the different treatments which indicated that the treatment is not toxic at the concentrations applied. However, more than 50% of the larvae had died in the infected groups 48 h *p*.*i*., regardless of the treatment which indicated that the treatment cannot protect the larvae against *B*. *cenocepacia* infection (Fig 4).

These observations were confirmed by the fact that no differences in CFU/larvae were observed among the different groups 24 h *p*.*i*. (S5 Fig).

### Evaluation of the combination tobramycin and miconazole in a mouse lung infection model

**Fig 5:**
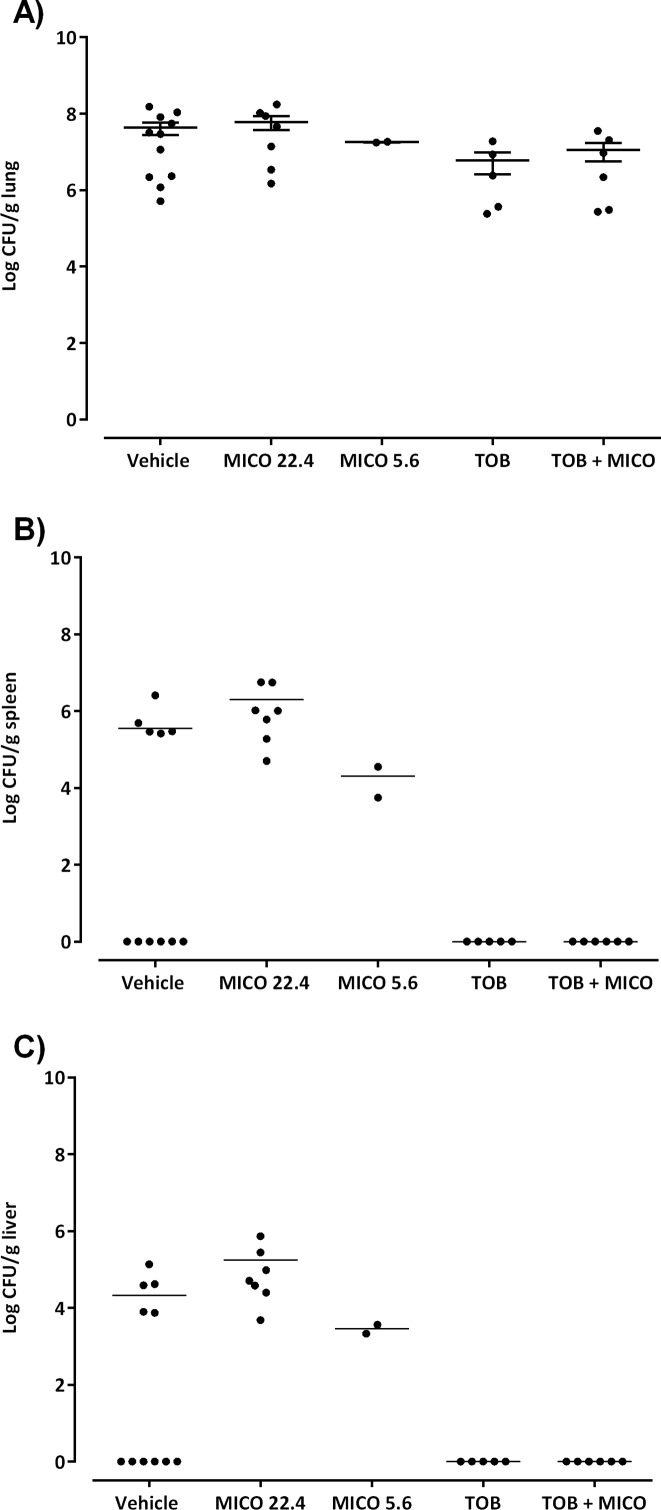
Effect of the combination therapy in a mouse lung infection model. Colony counts in lung (a), spleen (b), and liver (c) in mice treated with: (i) vehicle, (ii) 22.4 mg/kg bw miconazole (MICO), (iii) 5.6 mg/kg bw miconazole, (iv) 120 mg/kg bw tobramycin (TOB) and (v) 120 mg/kg bw tobramycin combined with 5.6 mg/kg bw miconazole (TOB + MICO). Error bars indicate SEM. Three independent repeats of mice treated with vehicle (n ≤ 3), two independent repeats of mice treated with MICO 22.4 mg/kg (n ≤ 3), one independent repeat of mice treated with MICO 5.6 mg/kg (n = 2), one independent repeat of mice treated with TOB 120 mg/kg (n = 6) and one independent repeat of mice treated with TOB + MICO (n = 6).

The combination of tobramycin and miconazole was evaluated in a *B*. *cenocepacia* mouse lung infection model. To this purpose, a formulation for inhalation was developed in which the particle size was lower than 5 μm (i.e., d_10_ = 0.23 μm, d_50_ = 0.41 μm and d_90_ = 3.68 μm), which is suitable for drug deposition in the lungs [25]. The formulation was found to be stable over 5 days (i.e. no shift in d_50_-value) which covered the duration of the *in vivo* study. The tobramycin concentration (120 mg/kg bw) used for the treatment of *B*. *cenocepacia* infected mice is about 129 times the concentration used in the MTP assay. The concentration of miconazole in the formulation was based on the ratio tobramycin/miconazole in the MTP experiments and the activity of the diluted formulation was confirmed against biofilms formed in 96-well MTPs (S6 Fig). Treatment of infected mice with tobramycin resulted in prevention of dissemination to liver and spleen, and a reduction in lung burden of approx. 1 log (Fig 5).

Mice treated with 22.4 mg/kg bw miconazole suffered from side effects and were characterized by a hunchback posture, weak breathing and an overall bad fur condition. Also dissemination of bacteria to liver and spleen was observed in every miconazole treated mouse, whereas only approx. half of the vehicle-treated mice (5/11) had dissemination of bacteria to liver and spleen (Fig 5). Therefore, a lower dose of miconazole (5.6 mg/kg bw) was chosen for the evaluation of the combination therapy in mice. As shown in Fig 5, no significant difference in lung burden could be observed between mice treated with tobramycin and tobramycin combined with miconazole. Also mice treated with this lower dose of miconazole alone suffered from the same toxic effects as 22.4 mg/kg bw miconazole.

## Discussion

### Library screening identifies antifungal imidazoles as antibacterial potentiators

Screening two repurposing libraries against *B*. *cenocepacia* biofilms in the presence of tobramycin resulted in the identification of 60 hit compounds. Four antifungal imidazoles, i.e. econazole, miconazole, oxiconazole, and ketoconazole were among the hits identified. Other azoles present in the library were less or not active, e.g. the triazoles fluconazole, itraconazole, or voriconazole. This is in accordance with results from a structure-activity relationship study on several oxiconazole derivatives in which compounds with an imidazole moiety were shown to be more potent antibacterials against *Klebsiella pneumoniae*, *Escherichia coli* and *Staphylococcus aureus* compared to triazoles [26]. The MIC of econazole and miconazole against *B*. *cenocepacia* exceeded 200 μM, indicating that they potentiate tobramycin at concentrations that do not inhibit growth of planktonic cells when administered alone. This is in accordance with a study in which no growth inhibitory activity was observed for miconazole against planktonic cells of *Pseudomonas aeruginosa* and *E*. *coli*, while the combination of miconazole with polymyxin B resulted in a strong synergistic interaction [27].

The activity of imidazoles towards Gram-positive bacteria has been described before [28–31]. Sud and Feingold investigated the bactericidal effect of miconazole and concluded that interference with the membrane of Gram-positive bacteria results in leakage of K^+^ [28]. In addition, imidazoles bind *S*. *aureus* flavohemoglobin, a protein with NO dioxygenase activity, causing nitrosative and oxidative stress [32]. Miconazole also binds flavohemoglobin of *E*. *coli*, and *in vitro* data showed that the combination of a NO-donor, miconazole and polymyxin B nonapeptide were effective to treat four ESBL-producing *E*. *coli* isolates [33].

### Activity of the combination in relevant model systems

Activity of tobramycin in combination with econazole or miconazole was tested in an *in vitro* 3D organotypic human cell culture model. 3D organotypic cell culture models are valuable research tools that mimic key aspects of the parental tissue and reduce the gap between *in vitro* cell culture models and physiological tissue [34,35]. A 3D A549 lung epithelial cell culture model has previously been developed and validated, and was used to study the colonization of *P*. *aeruginosa* and *Francisella tularensis*. These studies demonstrated that the 3D lung epithelial cells generated more *in vivo*-like phenotypes compared to conventional monolayers [20,36]. *B*. *cenocepacia* has been shown to both cause intracellular infections and form biofilms in the lungs of CF patients [8,37,38]. However, the biofilm formation capacity of *Burkholderia* species in CF lungs is still under debate [39]. Infection of the 3D lung epithelial model with *B*. *cenocepacia* K56-2 reflected aspects of the *in vivo* infection profile, as phenotypic characteristics that were indicative of both intracellular growth and biofilm formation were observed. In the absence of 3D cells, tobramycin showed an increased inhibition on biofilm formation in MTP in the presence of econazole or miconazole (Fig 3, left part), so the imidazoles tested both potentiate the biofilm-inhibiting and biofilm-eradicating activity of tobramycin. However, no potentiating effect could be observed when 3D long epithelial cells were present (Fig 3, right part).

The effect of the combination treatment was also evaluated in *G*. *mellonella*, which is a suitable model to test BCC virulence [40]. *G*. *mellonella* was used before to estimate the activity of treatments towards *B*. *cenocepacia* infections, including novel agents with potentiator activity [21]. Neither treatment with tobramycin, miconazole, econazole, nor the combination could protect the larvae against *B*. *cenocepacia* infection (Fig 4).

Finally, the combination of tobramycin and miconazole was evaluated in a *B*. *cenocepacia* mouse lung infection model. As 22.4 mg/kg bw miconazole demonstrated side effects, a lower dose of miconazole (5.6 mg/kg bw) was chosen for the evaluation of the combination therapy in mice. This dose is far below LD50 values for mice reported in literature, which range from 80 mg/kg for intravenous administration, to 220 mg/kg for intraperitoneal administration, and even up to 519 mg/kg upon oral administration [41]. However, also this lower dose appeared to be toxic for the mice used in the present study and no significant difference in lung burden could be observed between mice treated with tobramycin, and those treated with tobramycin and miconazole.

### Different activity in a conventional *in vitro* biofilm model system and other model systems of host-pathogen interactions

Based on *in vitro* data the promising combination of tobramycin with econazole or miconazole was evaluated in other model systems which all indicated that the combination did not improve the outcome upon infection with *B*. *cenocepacia* infection. Discrepancies in antimicrobial activity between *in vitro* and *in vivo* situations are often observed [42]. This may be because basic *in vitro* models for susceptibility testing lack host factors (e.g. cellular and humoral immunity of the host, the pathogen’s level of expression of virulence determinants and protein binding). Also, pharmacokinetic parameters as penetration into the site of infection are not taken into account. In the present study azoles lost their potentiating activity when they were investigated in models including host factors and it is thus likely that these host factors play a role in their inactivation. Protein binding of miconazole (reported to be 90%) might contribute to a substantial decrease in biologically active free fraction and loss of activity in the three models [43]. Also, a differential transcriptomic and phenotypic profile in the presence of host cells might result in a reduced susceptibility to the combination treatment.

The screening described in the present study was performed in one of the most frequently used models for the evaluation of novel biofilm inhibitory/eradication compounds, i.e. the static MTP biofilm model system [44]. Although this system has multiple advantages, it lacks many of the micro-environmental factors that are encountered during the natural course of infection of *B*. *cenocepacia*, including host components. Therefore, more advanced *in vitro* and *in vivo* model systems for screening of antibacterials should be considered that are more reflective of the host environment and thus may better predict the efficacy of the combination therapy. Of course, the suitability and predictive power of these model systems will need to be validated prior to use.

## Supporting information

S1 Table Results of the NIHCC 1&2 libraries screening for potentiators. The NIH Clinical Collection 1&2 were screened at a concentration of 100 μM in the presence of 512 μg/ml tobramycin for potentiators against biofilms of *B*. *cenocepacia* LMG 16656. Hits were subsequently tested in absence of tobramycin. Effect was evaluated using CTB staining. The values shown in the left column represent the mean residual metabolic activity of the compound in the presence of tobramycin compared to treatment with tobramycin alone, and the standard deviation. The values in the right column represent the mean residual metabolic activity of the compound compared to untreated control, and the standard deviation.
(DOCX)

S2 Table Overview of the hits identified after screening the NIH Clinical Collection 1&2 against biofilms of *B*. *cenocepacia*. Hits are classified according to their therapeutic indication. Compounds were tested at a concentration of 100 μM in the presence or absence of tobramycin (512 μg/ml). The values shown in the left column represent the mean residual metabolic activity of the compound in the presence of tobramycin compared to treatment with tobramycin alone, and the standard deviation. The values in the right column represent the mean residual metabolic activity of the compound compared to untreated control, and the standard deviation. X means that the compounds did not cause a significant difference in fluorescence signal compared to the signal of untreated biofilms. SNRI: serotonin-norepinephrine reuptake inhibitor, SSRI: serotonin reuptake inhibitor, TCA: tricyclic antidepressant.
(DOCX)

S1 Fig Number of CFU recovered from the biofilm, the supernatant, and the washing fluid, after treatment with econazole (100 μM), tobramycin (512 μg/ml), or the combination of both. Data shown are log CFU/biofilm or log CFU/ml (for supernatant or washing fluid). The data shown are the average of four biological replicates (error bars represent standard error).
(TIF)

S2 Fig Lactate dehydrogenase (LDH) release assay of 3D A549 lung epithelial cells that were non-infected and infected with *B*. *cenocepacia* K56-2 for 17h. LDH release is presented as a percentage of a positive control (3D lung epithelial cells lysed with Triton-X100). Data shown are average, error bars indicate SD.
(TIFF)

S3 Fig Evaluation of the treatments’ toxicity in the 3D cell culture model. Exposure of 3D A549 lung epithelial cells for 17 h to 10, 50, 100 μM econazole (B, E, G); 10, 50, 100 μM miconazole (C, F, H) or 1000 μg/mL tobramycin (D). A control condition where no antimicrobial agents were added is also included (A). High concentrations of the imidazoles resulted in detachment of host cells from microcarrier beads. Magnification is 300x. Scale bar is 400 μm.
(TIFF)

S4 Fig Number of CFU recovered from *B*. *cenocepacia* biofilm after treatment with varying concentrations of tobramycin (alone or combined). Biofilms of *B*. *cenocepacia* K56-2 were grown in 48-well MTP in the presence of tobramycin (T, in μg/ml) with 10μM econazole (E) or miconazole (M) to determine the optimal concentration for the tests in 3D human lung epithelial cells. (Data shown are average, n≥3, error bars indicate SD)
(TIF)

S5 Fig Determination of the bacterial load in *G*. *mellonella*. *G*. *mellonella* was infected with *B*. *cenocepacia* LMG 16656 and treated with tobramycin (TOB), econazole (ECO), or the combination. CFU/larvae was determined 24 h *p*.*i*. and treatment. Larvae were homogenized and plated on selective *Burkholderia* medium. (Data shown are average, n≥3, error bars indicate SEM).
(TIFF)

S6 Fig Evaluation of the *in vitro* effect of our formulation for inhalation. In order to evaluate the antimicrobial activity of a formulation for inhalation prior to use in the mouse lung infection model, biofilms of *B*. *cenocepacia* LMG 16656 were formed in 96-well MTPs and subsequently treated with the diluted (129x) formulation. This diluted formulation corresponds to vehicle (tween 80 dissolved in PS), 512 μg/mL tobramycin (TOB), 200 μM miconazole (MICO) or the combination of tobramycin and miconazole. The asterisk indicates a significantly different number in log CFU/BF compared to treatment with tobramycin alone (P value < 0.05). (Data shown are average, n≥3, error bars indicate SEM).
(TIFF)
