# Supplementary material for: Evaluation of combination therapy for Burkholderia cenocepacia lung infection in different in vitro and in vivo models
Source: PLoS One. 2017 Mar 1;12(3):e0172723. doi: 10.1371/journal.pone.0172723 (PMC5332113; doi:10.1371/journal.pone.0172723)
Supplement: S1 Table — The NIH Clinical Collection 1&2 were screened at a concentration of 100 μM in the presence of 512 μg/ml tobramycin for potentiators against biofilms of B. cenocepacia LMG 16656. Hits were subsequently tested in absence of tobramycin. Effect was evaluated using CTB staining. The values shown in the left column represent the mean residual metabolic activity of the compound in the presence of tobramycin compared to treatment with tobramycin alone, and the standard deviation. The values in the right column represent the mean residual metabolic activity of the compound compared to untreated control, and the standard deviation. (DOCX) [file pone.0172723.s001.docx]

**Table S1. The NIH Clinical Collection 1&2 were screened at a concentration of 100 μM in the presence of 512 μg/ml tobramycin for potentiators against biofilms of *B. cenocepacia* LMG 16656. Hits were subsequently tested in absence of tobramycin. Effect was evaluated using CTB staining. The values shown in the left column represent the mean residual metabolic activity of the compound in the presence of tobramycin compared to treatment with tobramycin alone, and the standard deviation. The values in the right column represent the mean residual metabolic activity of the compound compared to untreated control, and the standard deviation.**

| Pubchem code | Compound name | MEAN | SD | MEAN | SD |
| --- | --- | --- | --- | --- | --- |
| CPD000449281 | Nalbuphine | 30 | 14 |  |  |
| CPD000449275 | Raclopride | 50 | 24 |  |  |
| CPD000449271 | Zacopride | 58 | 40 |  |  |
| CPD000449276 | SKF 83566 D1 R agonist | 29 | 33 |  |  |
| CPD000449316 | 3'-deoxydenosine | 106 | 77 |  |  |
| CPD000449274 | AM 404 N-arachidonoylaminofenol | 7 | 9 | 99 | 10 |
| CPD000059053 | Pilocarpine | 114 | 29 |  |  |
| CPD000058291 | Nifedipine | 55 | 29 |  |  |
| CPD000042823 | Furbiprofen | 121 | 58 |  |  |
| CPD000059136 | Deferiprone | 106 | 15 |  |  |
| CPD000058470 | Loxapine | 2 | 5 | 43 | 6 |
| CPD000326694 | d-3-MEO-N-methylmorphinan | 39 | 24 |  |  |
| CPD000449282 | Duloxetine | 4 | 6 | 20 | 2 |
| CPD000449320 | Racecadotril | 217 | 97 |  |  |
| CPD000449318 | Benzene acetic acid | 53 | 12 |  |  |
| CPD000058345 | S-progesterone | 27 | 12 |  |  |
| CPD000058961 | Famotine | 73 | 22 |  |  |
| CPD000449299 | 5-HTR-3 agonist | 55 | 20 |  |  |
| CPD000466270 | Pancuronium | 52 | 13 |  |  |
| CPD000058175 | Metronidazole | 61 | 29 |  |  |
| CPD000449327 | Benzeneacetic acid | 67 | 25 |  |  |
| CPD000449323 | Benzeneacetonitrile | 23 | 4 |  |  |
| CPD000449328 | Benzeneethanaminde N alfa dimethyl | 57 | 8 |  |  |
| CPD000449294 | Zucapsaicin | 21 | 9 |  |  |
| CPD000058513 | Salbutamol | 69 | 19 |  |  |
| CPD000057879 | Vesamicol | 44 | 18 |  |  |
| CPD000469289 | Picrotin | 77 | 28 |  |  |
| CPD000449268 | Terazosin | 64 | 17 |  |  |
| CPD000449319 | Diphenylcyclopropenone | 26 | 20 |  |  |
| CPD000449326 | Thiazolidinecarboxylic acid | 75 | 40 |  |  |
| CPD000466274 | Mesoridazine | 23 | 5 |  |  |
| CPD000449313 | Pyridazinone difluoromethoxy | 58 | 40 |  |  |
| CPD000466275 | Phenothiazine | 17 | 10 |  |  |
| CPD000449322 | Cyclopentaquinolin | 48 | 10 |  |  |
| CPD000058306 | Clotrimazole | 25 | 3 |  |  |
| CPD000058255 | Loratadine | 12 | 10 |  |  |
| CPD000058500 | Phenelzine | 71 | 44 |  |  |
| CPD000449311 | Riluzole | 49 | 15 |  |  |
| CPD000449312 | Naltrindole | 22 | 10 |  |  |
| CPD000449277 | Nornicotine | 98 | 21 |  |  |
| CPD000449269 | Bifemelane | 6 | 2 | 23 | 9 |
| CPD000449284 | CGS antagonist D1/D2 receptors | 64 | 30 |  |  |
| CPD000449287 | Cinanserin | 17 | 8 |  |  |
| CPD000449272 | Cisapride | 75 | 56 |  |  |
| CPD000449273 | Indatrline | 12 | 11 |  |  |
| CPD000058520 | Trazodone | 37 | 15 |  |  |
| CPD000449301 | Prazosin | 75 | 55 |  |  |
| CPD000058525 | Urapidil | 123 | 43 |  |  |
| CPD000449278 | Cotinine | 70 | 26 |  |  |
| CPD000058313 | Cycloserine | 92 | 73 |  |  |
| CPD000466268 | Fluvoxamine | 13 | 12 |  |  |
| CPD000449270 | Doxepin | 11 | 4 |  |  |
| CPD000059133 | Trifluoperazine | 3 | 4 | 20 | 7 |
| CPD000058908 | Hydroxymethylmorphinan | 95 | 32 |  |  |
| CPD000449329 | Ornithine | 108 | 25 |  |  |
| CPD000148117 | Maprotiline | 12 | 11 |  |  |
| CPD000466272 | Pizotifen | 7 | 5 | 51 | 9 |
| CPD000059126 | Beta-estradiol | 30 | 13 |  |  |
| CPD000059046 | Diacetyl diaminohexane | 85 | 7 |  |  |
| CPD000058353 | Diphengydramine | 41 | 8 |  |  |
| CPD000449267 | Galanthamine | 100 | 26 |  |  |
| CPD000449296 | Ifenprodil | 20 | 14 |  |  |
| CPD000059171 | Tetraethylthiuram | 20 | 8 |  |  |
| CPD000449302 | Piribedil | 59 | 23 |  |  |
| CPD000058460 | Ketoconazole | 6 | 6 | 73 | 9 |
| CPD000058623 | Tripelennamine | 34 | 5 |  |  |
| CPD000449325 | Pyrazinecarboxamide | 90 | 29 |  |  |
| CPD000059105 | Amino tetrahydroacridine | 47 | 41 |  |  |
| CPD000058319 | Ethynylestradiol | 14 | 4 |  |  |
| CPD000449317 | Cytarabine | 102 | 25 |  |  |
| CPD000449324 | L-glutaminic acid | 63 | 15 |  |  |
| CPD000449305 | 16015-69-3 | 26 | 17 |  |  |
| CPD000449298 | Pramipexole | 103 | 34 |  |  |
| CPD000058189 | Lidocaine | 121 | 87 |  |  |
| CPD000449290 | indometacine | 197 | 62 |  |  |
| CPD000058555 | Iomelukast | 71 | 34 |  |  |
| CPD000466269 | Paroxetine maleate | 5 | 6 | 23 | 4 |
| CPD000449288 | Epigallocatechin | 189 | 42 |  |  |
| CPD000145728 | 5-amino salicylic acid | 120 | 53 |  |  |
| CPD000449321 | Etomoxir | 84 | 31 |  |  |
| CPD000338536 | Cephalexine | 51 | 13 |  |  |
| CPD000466390 | Pidotimod | 75 | 10 |  |  |
| CPD000466386 | Ramipril | 108 | 13 |  |  |
| CPD000469284 | Fenpiverinium | 66 | 20 |  |  |
| CPD000058610 | Nortestosterone | 40 | 1 |  |  |
| CPD000466384 | Nizatidine | 67 | 14 |  |  |
| CPD000059047 | Flucytosine | 81 | 13 |  |  |
| CPD000048684 | Oxcarbazepine | 67 | 39 |  |  |
| CPD000466385 | Troxipide | 60 | 4 |  |  |
| CPD000466341 | Actarit | 85 | 28 |  |  |
| CPD000469183 | Azelastine | 6 | 7 | 36 | 3 |
| CPD000466388 | Tocainide | 98 | 19 |  |  |
| CPD000499525 | Taxifolin | 62 | 12 |  |  |
| CPD000466387 | Levofloxacin | 89 | 8 |  |  |
| CPD000469182 | Cefatrizine | 82 | 17 |  |  |
| CPD000466364 | Idebenone | 31 | 14 |  |  |
| CPD000466366 | Levosulpiride | 81 | 38 |  |  |
| CPD000238142 | Pemoline | 92 | 22 |  |  |
| CPD000466343 | Letrozole | 89 | 18 |  |  |
| CPD000469184 | Meropenem | 75 | 20 |  |  |
| CPD000466339 | Orlistat | 88 | 29 |  |  |
| CPD000469179 | Ondansetran | 58 | 17 |  |  |
| CPD000059117 | Levonorgestrel | 98 | 14 |  |  |
| CPD000469197 | Cetraxate | 96 | 8 |  |  |
| CPD000149316 | Alprazolam | 81 | 6 |  |  |
| CPD000058464 | Lamotignine | 102 | 26 |  |  |
| CPD000059145 | Crotamiton | 73 | 27 |  |  |
| CPD000472526 | Amfebutamone | 89 | 21 |  |  |
| CPD000466340 | Alfuzonsine | 49 | 10 |  |  |
| CPD000449309 | Amisulpride | 56 | 11 |  |  |
| CPD000469292 | Lofepramine | 11 | 11 |  |  |
| CPD000466362 | Perospirone | 7 | 4 | 49 | 10 |
| CPD000059010 | Docetaxel | 25 | 18 |  |  |
| CPD000387107 | Honokiol | 5 | 4 | 8 | 3 |
| CPD000469196 | Tolterodine | 8 | 6 | 62 | 8 |
| CPD000466363 | Carmofur | 62 | 12 |  |  |
| CPD000469181 | N-methyl-paroxetine | 2 | 2 | 31 | 20 |
| CPD000466337 | Olmesartan | 84 | 47 |  |  |
| CPD000469593 | Potassium losartan | 66 | 24 |  |  |
| CPD000466338 | Temozolomide | 108 | 54 |  |  |
| CPD000058528 | Methyltestosterone | 55 | 43 |  |  |
| CPD000469195 | Tosufloxacin | 75 | 40 |  |  |
| CPD000466361 | Mecillinam | 62 | 12 |  |  |
| CPD000469177 | Atomoxetine | 5 | 6 | 47 | 7 |
| CPD000466336 | Artesunate | 52 | 25 |  |  |
| CPD000058959 | Itraconazole | 66 | 11 |  |  |
| CPD000469193 | Cefpodoxime | 68 | 13 |  |  |
| CPD000058803 | Buflomedil | 56 | 17 |  |  |
| CPD000012114 | Moclobemide | 71 | 16 |  |  |
| CPD000466330 | Halometasone | 51 | 24 |  |  |
| CPD000466357 | Triclabendazole | 43 | 15 |  |  |
| CPD000466331 | Rofecoxib | 27 | 5 |  |  |
| CPD000471619 | Bisoprolol | 69 | 10 |  |  |
| CPD000466334 | Ezetimibe | 43 | 18 |  |  |
| CPD000469176 | Tiagabine | 38 | 12 |  |  |
| CPD000466355 | Idarubicin | 11 | 8 |  |  |
| CPD000466360 | Flubendazole | 52 | 17 |  |  |
| CPD000466356 | Tacrolimus | 34 | 15 |  |  |
| CPD000469208 | Valaciclovir | 76 | 19 |  |  |
| CPD000466382 | Clarithromycin | 38 | 21 |  |  |
| CPD000466383 | Aripiprazole | 45 | 16 |  |  |
| CPD000471622 | Trimebutine | 44 | 10 |  |  |
| CPD000238198 | Mestalone | 47 | 4 |  |  |
| CPD000466370 | Nisoldipine | 62 | 19 |  |  |
| CPD000466371 | Piceid | 73 | 38 |  |  |
| CPD000149359 | Secnidazole | 74 | 22 |  |  |
| CPD000466369 | Nifekalant | 88 | 14 |  |  |
| CPD000466372 | Nateglinide | 96 | 19 |  |  |
| CPD000058691 | Megestrol acetaat | 41 | 16 |  |  |
| CPD000466374 | Ormetoprim | 110 | 31 |  |  |
| CPD000466377 | Zileuton | 51 | 11 |  |  |
| CPD000058350 | Stavudine | 74 | 44 |  |  |
| CPD000058918 | Gabexate | 68 | 21 |  |  |
| CPD000469293 | Oxiconazole | 4 | 4 | 65 | 16 |
| CPD000469235 | Kitasamycin | 42 | 13 |  |  |
| CPD000466375 | Famciclovir | 76 | 18 |  |  |
| CPD000326828 | Sotalol | 66 | 12 |  |  |
| CPD000466373 | Rufloxacin | 74 | 17 |  |  |
| CPD000466389 | Taxifolin | 90 | 21 |  |  |
| CPD000469211 | Alosetron | 86 | 21 |  |  |
| CPD000469159 | Fluticasone | 59 | 25 |  |  |
| CPD000469161 | Indinavir | 68 | 31 |  |  |
| CPD000469160 | Midazolam | 38 | 21 |  |  |
| CPD000466319 | Lamivudine | 77 | 17 |  |  |
| CPD000469151 | 366-70-1 | 117 | 31 |  |  |
| CPD000469280 | Esomeprazole | 34 | 18 |  |  |
| CPD000059146 | Sulfasalazine | 64 | 15 |  |  |
| CPD000466313 | Torasemide | 70 | 18 |  |  |
| CPD000469156 | Tropisetron | 37 | 15 |  |  |
| CPD000326795 | Ranolazine | 62 | 24 |  |  |
| CPD000058423 | Bupropion | 83 | 33 |  |  |
| CPD000471621 | Irsogladine | 53 | 27 |  |  |
| CPD000466376 | Acarbose | 79 | 13 |  |  |
| CPD000469294 | Benproperine | 2 | 4 | 25 | 8 |
| CPD000466378 | Fenprobamaat | 71 | 27 |  |  |
| CPD000058926 | Adamantan | 28 | 31 |  |  |
| CPD000449280 | Carvedilol | 11 | 12 |  |  |
| CPD000466379 | Lomifylline | 64 | 23 |  |  |
| CPD000466380 | Pazufloxacin | 69 | 20 |  |  |
| CPD000466381 | Miglitol | 76 | 44 |  |  |
| CPD000058373 | Tranilast | 59 | 35 |  |  |
| CPD000466345 | Olanzapine | 16 | 19 |  |  |
| CPD000449297 | Nefazodone HCl | 10 | 14 | 47 | 22 |
| CPD000469185 | Moxifloxacin | 49 | 28 |  |  |
| CPD000469186 | Nelfinavir | 52 | 18 |  |  |
| CPD000469187 | Pravastatin | 143 | 52 |  |  |
| CPD000466344 | Topotecan | 79 | 36 |  |  |
| CPD000466303 | Levetiracetam | 79 | 21 |  |  |
| CPD000469142 | Pramipexole | 63 | 23 |  |  |
| CPD000466323 | Risperidone | 22 | 10 |  |  |
| CPD000469167 | Pioglitazone | 73 | 31 |  |  |
| CPD000469147 | Cilastatin | 66 | 24 |  |  |
| CPD000466348 | Argatroban | 74 | 35 |  |  |
| CPD000466327 | Valdecoxib | 59 | 19 |  |  |
| CPD000466346 | Naftopidil | 5 | 6 | 24 | 5 |
| CPD000156231 | Nobiletin | 29 | 24 |  |  |
| CPD000466304 | Finasteride | 76 | 34 |  |  |
| CPD000469145 | Zolpidem | 81 | 29 |  |  |
| CPD000048458 | Viramune | 117 | 66 |  |  |
| CPD000466325 | Topiramate | 123 | 41 |  |  |
| CPD000466350 | Voriconazole | 94 | 29 |  |  |
| CPD000469190 | Fenoldopam | 94 | 39 |  |  |
| CPD000471612 | Rosiglitazone | 51 | 17 |  |  |
| CPD000469191 | Escitalopram | 22 | 14 |  |  |
| CPD000058866 | Zeranol | 58 | 20 |  |  |
| CPD000466354 | Latanoprost | 17 | 13 |  |  |
| CPD000058576 | Didanosine | 90 | 18 |  |  |
| CPD000466298 | Sertraline | 5 | 7 | 2 | 4 |
| CPD000466353 | Calcipotriol | 38 | 10 |  |  |
| CPD000466308 | Epirubicin | 35 | 7 |  |  |
| CPD000466329 | Bicalutamide | 41 | 21 |  |  |
| CPD000469192 | Benidipine | 56 | 9 |  |  |
| CPD000466352 | Amlexanox | 79 | 13 |  |  |
| CPD000469148 | Cerivastatin | 109 | 28 |  |  |
| CPD000466309 | Icariin | 101 | 19 |  |  |
| CPD000466310 | Methylandrosternediol | 109 | 16 |  |  |
| CPD000466307 | Triptolide | 103 | 20 |  |  |
| CPD000469170 | Rosiglitazone | 88 | 19 |  |  |
| CPD000059106 | Tegafur | 79 | 13 |  |  |
| CPD000466392 | Oligomycin | 15 | 7 |  |  |
| CPD000469199 | Benazepril | 93 | 16 |  |  |
| CPD000058877 | Oxymetholone | 41 | 15 |  |  |
| CPD000059060 | Ipriflavone | 75 | 16 |  |  |
| CPD000058286 | Oxaprozin | 74 | 16 |  |  |
| CPD000058510 | Rolipram | 92 | 31 |  |  |
| CPD000469200 | Mosapride | 75 | 28 |  |  |
| CPD000466391 | Isoquercitrin | 93 | 25 |  |  |
| CPD000058450 | Flumazenil | 107 | 10 |  |  |
| CPD000469164 | Ozagrel | 127 | 32 |  |  |
| CPD000466394 | Hyperoside | 123 | 26 |  |  |
| CPD000466322 | Rifabutin | 27 | 20 |  |  |
| CPD000469141 | Esmolol | 72 | 21 |  |  |
| CPD000466321 | Tadalafil | 22 | 14 |  |  |
| CPD000058957 | Modafinil | 31 | 20 |  |  |
| CPD000058570 | Doxorubicin | 18 | 16 |  |  |
| CPD000469209 | Moxonidine | 74 | 58 |  |  |
| CPD000058302 | Nitrazepam | 48 | 40 |  |  |
| CPD000387024 | Pefloxacin | 105 | 88 |  |  |
| CPD000469154 | Venlafaxine | 77 | 44 |  |  |
| CPD000469592 | Pantoprazole | 49 | 50 |  |  |
| CPD000058366 | Nitrendipine | 8 | 4 | 73 | 10 |
| CPD000469290 | Saquinavir | 11 | 7 |  |  |
| CPD000058970 | Bifonazole | 12 | 10 |  |  |
| CPD000469158 | Sumatriptan | 47 | 16 |  |  |
| CPD000466314 | Exemestane | 31 | 5 |  |  |
| CPD000466367 | Nitazoxanide | 6 | 4 | 16 | 13 |
| CPD000058398 | Diazepam | 25 | 15 |  |  |
| CPD000471623 | Quetiapine | 25 | 13 |  |  |
| CPD000112560 | Rutin | 63 | 18 |  |  |
| CPD000466317 | Penciclovir | 60 | 11 |  |  |
| CPD000466393 | Calcitriol | 18 | 11 |  |  |
| CPD000469140 | Diphenoxylate | 76 | 39 |  |  |
| CPD000449307 | Felbamate | 73 | 26 |  |  |
| CPD000058855 | Droperidol | 9 | 7 | 62 | 10 |
| CPD000035998 | Pentoxifylline | 79 | 17 |  |  |
| CPD000058461 | Ketorolac | 45 | 21 |  |  |
| CPD000466395 | Ritonavir | 32 | 7 |  |  |
| CPD000469210 | Vinorelbine tartrate | 5 | 3 | 22 | 9 |
| CPD000466335 | Linezolid | 56 | 41 |  |  |
| CPD000469203 | Lomerizine | 38 | 11 |  |  |
| CPD000466351 | Efavirenz | 2 | 2 | 20 | 8 |
| CPD000466306 | Irbesartan | 55 | 34 |  |  |
| CPD000466305 | Repaglinide | 64 | 14 |  |  |
| CPD000238204 | Ethylestrenol | 6 | 7 | 46 | 9 |
| CPD000440694 | Pterostilbene | 5 | 9 | 30 | 10 |
| CPD000469144 | Roxatidine | 88 | 37 |  |  |
| CPD000471616 | Dexbrompheniramine | 37 | 62 |  |  |
| CPD000469168 | Anagrelide | 47 | 32 |  |  |
| CPD000471618 | Tegaserod | 4 | 5 | 10 | 4 |
| CPD000058475 | Milrinone | 78 | 21 |  |  |
| CPD000466315 | Levocetirizine | 83 | 16 |  |  |
| CPD000326936 | Citalopram | 27 | 11 |  |  |
| CPD000048468 | Ticlopidine | 18 | 9 |  |  |
| CPD000469165 | Sodium loxoprofen | 92 | 18 |  |  |
| CPD000466316 | Zafirlukast | 23 | 13 |  |  |
| CPD000469152 | Terbinafine | 45 | 26 |  |  |
| CPD000466320 | Isradipine | 23 | 18 |  |  |
| CPD000466318 | Valsartan | 91 | 17 |  |  |
| CPD000449291 | Piroxicam | 76 | 23 |  |  |
| CPD000469282 | Glycopyrrolate | 94 | 19 |  |  |
| CPD000449286 | Physostigmine | 62 | 20 |  |  |
| CPD000058465 | Lobeline | 40 | 30 |  |  |
| CPD000058436 | Doxylamine | 52 | 10 |  |  |
| CPD000449266 | Milnacipran | 75 | 26 |  |  |
| CPD000449315 | Fluoropyrimidone | 80 | 24 |  |  |
| CPD000466271 | Chlorpheniramine | 24 | 10 |  |  |
| CPD000466333 | Dofetilide | 110 | 38 |  |  |
| CPD000471620 | Formoterol | 71 | 9 |  |  |
| CPD000525252 | Rizatriptan | 95 | 20 |  |  |
| CPD000466332 | Rifapentine | 10 | 6 | 35 | 8 |
| CPD000469178 | Loteprednol | 97 | 21 |  |  |
| CPD000466359 | Enalaprilat | 107 | 15 |  |  |
| CPD000449292 | Donepezil | 5 | 4 | 57 | 5 |
| CPD000238177 | Nimetazepan | 79 | 15 |  |  |
| CPD000466365 | Nicorandil | 156 | 39 |  |  |
| CPD000466326 | Telmisartan | 61 | 7 |  |  |
| CPD000469143 | Itopride | 50 | 21 |  |  |
| CPD000466324 | Rifaximin | 13 | 15 |  |  |
| CPD000469188 | Montelukast | 8 | 5 | 85 | 21 |
| CPD000058253 | Didezoxycytidine | 45 | 8 |  |  |
| CPD000466276 | Imidazol amine | 51 | 27 |  |  |
| CPD000466280 | Pyridobenzodiazepin | 45 | 40 |  |  |
| CPD000466278 | MK 886 | 12 | 7 |  |  |
| CPD000466277 | Imidazol carboxylic | 37 | 11 |  |  |
| CPD000466281 | Acetamide | 66 | 49 |  |  |
| CPD000466283 | Altanserin | 42 | 10 |  |  |
| CPD000058420 | Betaxolol | 67 | 31 |  |  |
| CPD000466311 | Indirubine | 78 | 32 |  |  |
| CPD000466285 | Azasetron | 52 | 9 |  |  |
| CPD000466287 | GR 89696 | 54 | 15 |  |  |
| CPD000058773 | Delta hydrocortisone | 83 | 13 |  |  |
| CPD000058392 | Diazoxide | 65 | 14 |  |  |
| CPD000058612 | Chloroadenosine | 80 | 25 |  |  |
| CPD000058726 | Ornidazole | 91 | 21 |  |  |
| CPD000058572 | DMPP | 108 | 26 |  |  |
| CPD000058507 | Pirenperone | 29 | 8 |  |  |
| CPD000059128 | Mestramol | 68 | 26 |  |  |
| CPD000059100 | Aminoethyl pyridine | 87 | 27 |  |  |
| CPD000059142 | Benactyzine | 125 | 14 |  |  |
| CPD000059158 | Dichloroacetic acid | 97 | 18 |  |  |
| CPD000059165 | Bestatin | 75 | 16 |  |  |
| CPD000469213 | Toreminfene | 19 | 1 |  |  |
| CPD000469214 | Goserelin | 38 | 13 |  |  |
| CPD000469212 | Secoisolarciciresinol | 66 | 11 |  |  |
| CPD000469217 | Raltitrexed | 90 | 25 |  |  |
| CPD000469229 | Doxapram | 79 | 25 |  |  |
| CPD000466294 | Serotonin agonist | 48 | 4 |  |  |
| CPD000112281 | Brucine | 68 | 8 |  |  |
| CPD000059115 | Tryptoline | 37 | 8 |  |  |
| CPD000058411 | Fluphenazine | 5 | 4 | 26 | 8 |
| CPD000469233 | Palonosetron | 18 | 5 |  |  |
| CPD000058746 | Naproxen | 96 | 20 |  |  |
| CPD000058904 | Mepivacaine | 105 | 14 |  |  |
| CPD000058310 | Dibromohydroxybenz | 29 | 31 |  |  |
| CPD000058300 | Nimodipine | 21 | 5 |  |  |
| CPD000058701 | Rolitetracycline | 60 | 8 |  |  |
| CPD000058715 | Epirizole | 63 | 9 |  |  |
| CPD000058273 | Azauridine | 72 | 8 |  |  |
| CPD000466922 | Reichsteins | 53 | 17 |  |  |
| CPD000059086 | Pyridinemethanol | 93 | 12 |  |  |
| CPD000449283 | Haloperidol | 6 | 1 | 20 | 3 |
| CPD000449279 | Stiripentol | 62 | 14 |  |  |
| CPD000449303 | Fluperlapine | 41 | 10 |  |  |
| CPD000058660 | Oxyphenomium | 57 | 10 |  |  |
| CPD000112358 | Homoveratrylamine | 90 | 10 |  |  |
| CPD000058194 | Tinidalzole | 104 | 27 |  |  |
| CPD000058741 | Xanthinol | 121 | 32 |  |  |
| CPD000059111 | Synephrine | 116 | 36 |  |  |
| CPD000058206 | Resveratrol | 56 | 10 |  |  |
| CPD000059093 | Maltol | 71 | 10 |  |  |
| CPD000059077 | Aminoimidazole | 52 | 6 |  |  |
| CPD000059011 | Enrofloxacin | 61 | 12 |  |  |
| CPD000058603 | Dehydrocholic acid | 72 | 23 |  |  |
| CPD000058250 | Cefaclor | 88 | 14 |  |  |
| CPD000059044 | Benzylimidazole | 90 | 20 |  |  |
| CPD000469136 | Duloxetine HCl | 6 | 2 | 10 | 4 |
| CPD000469155 | Vardenafil | 90 | 15 |  |  |
| CPD000469137 | Ropivacaine | 84 | 14 |  |  |
| CPD000466301 | Anastrozole | 97 | 15 |  |  |
| CPD000058462 | Ketotifen | 70 | 8 |  |  |
| CPD000058769 | Medroxy progesterone | 94 | 14 |  |  |
| CPD000466919 | Pinacidil | 124 | 10 |  |  |
| CPD000058266 | Nitro-indazole | 66 | 17 |  |  |
| CPD000112269 | Methoxytryptamine | 67 | 17 |  |  |
| CPD000059045 | Phenothiazine | 18 | 4 |  |  |
| CPD000058553 | Cladribine | 81 | 11 |  |  |
| CPD000469138 | Granisetron hydrochloride | 45 | 8 |  |  |
| CPD000466293 | Rimcazole | 13 | 10 |  |  |
| CPD000466292 | Nafadoride | 11 | 6 |  |  |
| CPD000058856 | Desoximetasone | 62 | 18 |  |  |
| CPD000471617 | Dexchlorpheniramine | 58 | 13 |  |  |
| CPD000466288 | Guanidine | 104 | 17 |  |  |
| CPD000466290 | Smr000466290 | 18 | 7 |  |  |
| CPD000466284 | AM251 | 93 | 25 |  |  |
| CPD000466289 | HTMT | 98 | 35 |  |  |
| CPD000466286 | Benzo phenanthridine | 53 | 27 |  |  |
| CPD000466291 | Methanesulfonamide | 66 | 15 |  |  |
| CPD000466279 | H2-indol-2-one | 66 | 9 |  |  |
| CPD000466920 | Beclosmethasone | 64 | 17 |  |  |
| CPD000058847 | Omeprazole | 56 | 17 |  |  |
| CPD000469228 | Dolastron | 49 | 16 |  |  |
| CPD000449310 | Zolmitriptan | 68 | 23 |  |  |
| CPD000469223 | Tremaulacin | 72 | 33 |  |  |
| CPD000469227 | Dactinomycine | 42 | 12 |  |  |
| CPD000449308 | Tramadol | 69 | 24 |  |  |
| CPD000469226 | Chlordiazeposide | 66 | 25 |  |  |
| CPD000469225 | Cefixime | 101 | 20 |  |  |
| CPD000469224 | Cefdinir | 95 | 22 |  |  |
| CPD000469232 | Lofexidine | 67 | 24 |  |  |
| CPD000469221 | Balsalazide | 84 | 10 |  |  |
| CPD000469220 | Olopatadine | 27 | 20 |  |  |
| CPD000469287 | Itavastatin | 57 | 31 |  |  |
| CPD000058334 | Cortisone | 61 | 35 |  |  |
| CPD000058431 | Cyproheptadine | 28 | 27 |  |  |
| CPD000469230 | Homoharringt onine | 46 | 10 |  |  |
| CPD000058318 | Corticosterone | 65 | 39 |  |  |
| CPD000471625 | Vecuronium | 44 | 28 |  |  |
| CPD000469219 | Itibolone | 58 | 37 |  |  |
| CPD000058212 | Niacinamide | 102 | 46 |  |  |
| CPD000059131 | Nialamide | 100 | 46 |  |  |
| CPD000469153 | Vindesine | 45 | 9 |  |  |
| CPD000058540 | Vincristine | 68 | 21 |  |  |
| CPD000466342 | Lacidipine | 90 | 18 |  |  |
| CPD000466347 | Mirtazapine | 58 | 10 |  |  |
| CPD000469285 | Ampiroxicam | 78 | 4 |  |  |
| CPD000466368 | Glimepiride | 85 | 23 |  |  |
| CPD000469198 | Amlodipine | 12 | 2 |  |  |
| CPD000469174 | Rabeprazole | 51 | 13 |  |  |
| CPD000058704 | Clofazimine | 87 | 14 |  |  |
| CPD000469166 | Irinotecan | 76 | 17 |  |  |
| CPD000058469 | Lansoprazole | 71 | 13 |  |  |
| CPD000149358 | Desloratadine | 24 | 9 |  |  |
| CPD000058772 | Premarine | 96 | 30 |  |  |
| CPD000058481 | Mifepristone | 111 | 23 |  |  |
| CPD000112002 | Etoposide | 79 | 6 |  |  |
| CPD000238156 | Sibutramine | 81 | 16 |  |  |
| CPD000469632 | Clobenpropit | 64 | 15 |  |  |
| CPD000469231 | Huperzine | 81 | 9 |  |  |
| CPD000472527 | Sibutraminde | 79 | 16 |  |  |
| CPD000058410 | Lorazepam | 71 | 25 |  |  |
| CPD000469633 | Azaspiro-decane-dione | 72 | 12 |  |  |
| CPD000469631 | Adenosine | 82 | 13 |  |  |
| CPD000058296 | Amiodarone | 84 | 11 |  |  |
| CPD000336944 | Mevastatine | 80 | 8 |  |  |
| CPD000469175 | Imatinib | 45 | 10 |  |  |
| CPD000468736 | Methylperon | 65 | 12 |  |  |
| CPD000469594 | Parecoxib | 82 | 7 |  |  |
| CPD000058504 | Pergolide | 106 | 8 |  |  |
| CPD000471626 | Atracurium | 70 | 15 |  |  |
| CPD000469218 | Artemether | 95 | 15 |  |  |
| CPD000058445 | Ebselen | 64 | 18 |  |  |
| CPD000468733 | 12066B | 14 | 10 |  |  |
| CPD000469222 | Teletromycine | 72 | 10 |  |  |
| CPD000468732 | CCPA | 97 | 24 |  |  |
| CPD000468734 | Methanone | 83 | 22 |  |  |
| CPD000058878 | Stanzolol | 99 | 14 |  |  |
| CPD000238180 | Zaleplon | 73 | 9 |  |  |
| CPD000112594 | Prostaglandin | 87 | 6 |  |  |
| CPD000058344 | Testosteron | 78 | 18 |  |  |
| CPD000059075 | Dehydroepiandrosterone | 79 | 6 |  |  |
| CPD000466297 | - | 34 | 5 |  |  |
| CPD000466299 | Dup 697 | 112 | 17 |  |  |
| CPD000466300 | Nonyloxytryptamine | 14 | 6 |  |  |
| CPD000466295 | Salmeterol | 37 | 6 |  |  |
| CPD000466296 | SB 205607 | 67 | 14 |  |  |
| CPD000326935 | R-SCH-23390 | 71 | 25 |  |  |
| CPD000058230 | Doxepin | 2 | 1 | 39 | 7 |
| CPD000058382 | Dipyridamole | 36 | 12 |  |  |
| CPD000059151 | Propofol | 38 | 5 |  |  |
| CPD000058600 | Ethacrynic acid | 20 | 13 |  |  |
| CPD000058187 | Flutamide | 6 | 2 | 36 | 6 |
| CPD000058299 | Fenofibrate | 50 | 35 |  |  |
| CPD000058202 | Furosemide | 33 | 9 |  |  |
| CPD000038082 | Fluorouracil | 33 | 14 |  |  |
| CPD000471860 | Folic acid | 50 | 16 |  |  |
| CPD000653523 | Hydrocortisone | 42 | 12 |  |  |
| CPD000653536 | Cortell | 45 | 5 |  |  |
| CPD000058184 | Ibuprofen | 55 | 20 |  |  |
| CPD000040181 | Ketoprofen | 52 | 13 |  |  |
| CPD001906766 | Minocycline | 32 | 3 |  |  |
| CPD000058733 | Miconazole | 1 | 1 | 49 | 11 |
| CPD000059134 | Metyrapone | 50 | 16 |  |  |
| CPD001317860 | Pyrogluatmic acid | 82 | 35 |  |  |
| CPD000058975 | Nadolol | 68 | 25 |  |  |
| CPD000058999 | Disipal | 17 | 15 |  |  |
| CPD000058192 | Ofloxacin | 87 | 47 |  |  |
| CPD000059120 | Pindolol | 60 | 27 |  |  |
| CPD000037139 | Praziquantel | 40 | 21 |  |  |
| CPD000059104 | Phenylbutyric acid | 87 | 57 |  |  |
| CPD000058326 | Prednisolone | 61 | 19 |  |  |
| CPD000058379 | Promethazine | 4 | 8 | 30 | 10 |
| CPD000058180 | Perphenazine | 3 | 3 | 46 | 8 |
| CPD000718761 | Prednisolone | 58 | 20 |  |  |
| CPD000058506 | Prilocaine | 104 | 19 |  |  |
| CPD001227202 | Prednisone | 65 | 27 |  |  |
| CPD000059161 | DL-penicillamine | 88 | 28 |  |  |
| CPD000058579 | Piperacilline | 76 | 25 |  |  |
| CPD000857275 | Quinidine | 28 | 5 |  |  |
| CPD000653467 | Ranitidine | 47 | 19 |  |  |
| CPD001906767 | Rifampicine | 17 | 3 |  |  |
| CPD000058245 | Retinoic acid | 23 | 11 |  |  |
| CPD000471892 | Spironolactone | 34 | 13 |  |  |
| CPD000035999 | Trimethoprim | 90 | 25 |  |  |
| CPD000058219 | Tyzine | 91 | 13 |  |  |
| CPD000059176 | Thyroxine | 56 | 13 |  |  |
| CPD000058515 | Trihexyphenidyl | 20 | 9 |  |  |
| CPD000058403 | Ursodeoxycholic acid | 97 | 23 |  |  |
| CPD000059064 | Dapsone | 105 | 26 |  |  |
| CPD001370746 | Symmertrel | 58 | 15 |  |  |
| CPD000058849 | Warfarin | 74 | 7 |  |  |
| CPD000058394 | Acetazolamide | 78 | 21 |  |  |
| CPD000059083 | Allopurinol | 67 | 11 |  |  |
| CPD001906768 | Atropine | 76 | 17 |  |  |
| CPD000058264 | Nalicixic acid | 57 | 21 |  |  |
| CPD001567029 | Triiodothyronine | 21 | 6 |  |  |
| CPD000058284 | Hydroflumethiazide | 79 | 18 |  |  |
| CPD000058368 | Amitryptiline | 0 | 0 | 22 | 4 |
| CPD000058613 | Busulfan | 71 | 22 |  |  |
| CPD000058269 | Chlorzoxazone | 84 | 19 |  |  |
| CPD000058429 | Chlorothiazide | 91 | 27 |  |  |
| CPD001370748 | Cimetidine | 107 | 30 |  |  |
| CPD000058433 | Carisoprodol | 103 | 42 |  |  |
| CPD000058364 | Chlorpropamide | 104 | 24 |  |  |
| CPD000058440 | Dicyclomine | 24 | 14 |  |  |
| CPD000312779 | Chloroxine | 5 | 6 | 44 | 11 |
| CPD000058723 | Diflunisal | 63 | 15 |  |  |
| CPD001370749 | Econazole | 1 | 1 | 21 | 5 |
| CPD001370750 | Ethionamide | 39 | 24 |  |  |
| CPD000058719 | Methocarbamol | 68 | 22 |  |  |
| CPD000035778 | Hydrochlorothiazide | 70 | 22 |  |  |
| CPD001370751 | Vistaril | 25 | 3 |  |  |
| CPD000058356 | Hexachlorophene | 0 | 1 | 5 | 6 |
| CPD000059082 | Isoniazid | 73 | 26 |  |  |
| CPD000058729 | Duvadilan | 111 | 47 |  |  |
| CPD000058267 | Isoproterenol | 89 | 30 |  |  |
| CPD000471847 | Triclosan | 3 | 3 | 1 | 1 |
| CPD000058188 | Mefenamic acid | 60 | 32 |  |  |
| CPD000058832 | Cantil | 72 | 13 |  |  |
| CPD000058471 | Metoclopramide | 57 | 23 |  |  |
| CPD001370753 | Methyldopa | 55 | 22 |  |  |
| CPD000058271 | Nitrofurantoin | 52 | 24 |  |  |
| CPD000058486 | Nortriptyline | 2 | 3 | 21 | 3 |
| CPD000058292 | Naphazoline | 28 | 6 |  |  |
| CPD000059024 | Nicotinic | 50 | 20 |  |  |
| CPD000058817 | Norflex | 16 | 10 |  |  |
| CPD001614498 | Oxytetracycline | 39 | 13 |  |  |
| CPD000718771 | Procaine | 97 | 38 |  |  |
| CPD000058714 | Pyrimethamine | 54 | 20 |  |  |
| CPD000058661 | Propantheline bromide | 39 | 11 |  |  |
| CPD000058280 | Probenecid | 100 | 96 |  |  |
| CPD001906769 | Pyridine 2 aldoxime | 117 | 39 |  |  |
| CPD000058501 | Primidone | 59 | 38 |  |  |
| CPD000058275 | Propylthiouracil | 48 | 11 |  |  |
| CPD000036662 | Pyrazinamide | 72 | 20 |  |  |
| CPD000059079 | Probesyl | 81 | 54 |  |  |
| CPD000037657 | Sulfisoaxole | 76 | 48 |  |  |
| CPD000058223 | Sulfamethoxaole | 113 | 61 |  |  |
| CPD000058173 | Sulfacetamide | 153 | 78 |  |  |
| CPD000058991 | Sulfinpyrazone | 118 | 45 |  |  |
| CPD000326718 | Sulindac | 121 | 52 |  |  |
| CPD001906770 | Tetracycline | 53 | 32 |  |  |
| CPD000058537 | Theophylline | 97 | 62 |  |  |
| CPD000058363 | Tolbutamide | 58 | 12 |  |  |
| CPD000059118 | Triamteren | 44 | 11 |  |  |
| CPD000059081 | Intropin | 65 | 31 |  |  |
| CPD000058416 | Amoxapine | 5 | 6 | 34 | 5 |
| CPD000471872 | Adenine | 65 | 14 |  |  |
| CPD000036768 | Atenolol | 68 | 34 |  |  |
| CPD001491671 | Tamoxifen | 4 | 9 | 69 | 10 |
| CPD000058418 | Bumetanide | 57 | 17 |  |  |
| CPD000058745 | Clobetasol | 44 | 9 |  |  |
| CPD000058254 | Chlorpromazine | 2 | 4 | 22 | 10 |
| CPD001491644 | Cefazoline sodium | 75 | 18 |  |  |
| CPD000059061 | Captopril | 141 | 41 |  |  |
| CPD000058372 | Chlorambucil | 136 | 39 |  |  |
| CPD000058809 | Cefoxitin | 76 | 43 |  |  |
| CPD000058321 | Danazol | 96 | 32 |  |  |
| CPD000058375 | Diltiazem | 52 | 12 |  |  |
| CPD001906774 | Digoxin | 170 | 37 |  |  |
| CPD000058346 | 17- beta estradiol | 54 | 27 |  |  |
| CPD000058672 | Edroponium | 86 | 15 |  |  |
| CPD000058329 | Fluocinolone acetonide | 67 | 14 |  |  |
| CPD000042823 | Flurbiprofen | 101 | 17 |  |  |
| CPD000058455 | Glipizide | 111 | 28 |  |  |
| CPD000058393 | Gemfibrozil | 127 | 45 |  |  |
| CPD000058229 | Glyburide | 99 | 21 |  |  |
| CPD000058328 | hydrocortisone | 146 | 43 |  |  |
| CPD000058829 | Indapamide | 178 | 47 |  |  |
| CPD001906775 | Ipratropium bromide | 135 | 54 |  |  |
| CPD000058388 | Imipramine | 4 | 6 | 36 | 7 |
| CPD000058463 | Labetalol | 117 | 32 |  |  |
| CPD000058466 | Loperamide | 2 | 4 | 36 | 6 |
| CPD000058833 | Pro-amatine | 103 | 34 |  |  |
| CPD000653524 | Medroxyprogesterone acetate | 18 | 12 |  |  |
| CPD001906776 | 19-norethindrone acetate | 24 | 12 |  |  |
| CPD000499579 | 19-norethindrone | 76 | 19 |  |  |
| CPD000059074 | Nicotine | 99 | 27 |  |  |
| CPD001456372 | Cardene | 27 | 11 |  |  |
| CPD000058835 | Nabmetone | 99 | 3 |  |  |
| CPD000058490 | Oxybutynin | 8 | 9 | 44 | 9 |
| CPD000058605 | Mestinon | 99 | 16 |  |  |
| CPD001453705 | Rythmol | 12 | 4 |  |  |
| CPD001491654 | Pfizerpen | 68 | 26 |  |  |
| CPD000499581 | Valproic acid | 75 | 34 |  |  |
| CPD000058821 | Pro cyclidine | 6 | 7 | 48 | 20 |
| CPD000875264 | Proxymetacaine | 43 | 17 |  |  |
| CPD000058766 | Naloxone | 66 | 34 |  |  |
| CPD001906777 | Spectinomycin | 52 | 18 |  |  |
| CPD000058523 | Tropicamide | 65 | 26 |  |  |
| CPD000058290 | Tolazamide | 94 | 58 |  |  |
| CPD000058335 | Triamcinolone acetonide | 49 | 12 |  |  |
| CPD001456519 | Timolol | 67 | 17 |  |  |
| CPD000058170 | Thiabendazole | 63 | 18 |  |  |
| CPD000058380 | Thioridazine | 0 | 1 | 18 | 3 |
| CPD000058181 | Altretamine | 60 | 21 |  |  |
| CPD001491672 | Phylloquinone | 84 | 15 |  |  |
| CPD001491659 | Eryped | 204 | 131 |  |  |
| CPD000058422 | Dibenzyline | 12 | 11 |  |  |
| CPD000058693 | Medrysone | 38 | 26 |  |  |
| CPD000058524 | Thalidomide | 80 | 67 |  |  |
| CPD000857229 | Aminolevulinec | 74 | 44 |  |  |
| CPD001496929 | Carbinoxamine | 51 | 45 |  |  |
| CPD001496930 | Demeclocycline | 57 | 29 |  |  |
| CPD001496932 | Westcort | 54 | 28 |  |  |
| CPD000449328 | Selegiline | 62 | 23 |  |  |
| CPD000058840 | 6-2-ethoxy-1-naphthamido | 56 | 20 |  |  |
| CPD000875314 | Primaquine | 7 | 5 | 29 | 2 |
| CPD001496934 | Micropenin | 94 | 15 |  |  |
| CPD001550033 | Doxycycline | 43 | 20 |  |  |
| CPD001233361 | Beclomethasone | 50 | 13 |  |  |
| CPD000058721 | Cromolyn | 94 | 11 |  |  |
| CPD000149600 | Priscoline | 43 | 16 |  |  |
| CPD000544948 | Mercaptopurine | 90 | 18 |  |  |
| CPD000427366 | Azathioprine | 98 | 40 |  |  |
| CPD000036735 | Albendazole | 88 | 25 |  |  |
| CPD000718755 | Griseofulvin | 69 | 26 |  |  |
| CPD000059006 | Lincomycine | 88 | 39 |  |  |
| CPD001496938 | Methazol amide | 107 | 43 |  |  |
| CPD001496939 | Terbutaline | 83 | 44 |  |  |
| CPD000471888 | Mypirocin | 122 | 70 |  |  |
| CPD000058331 | Fluocinonide | 64 | 10 |  |  |
| CPD000875233 | Mefloquine hcl | 4 | 4 | 11 | 8 |
| CPD001496941 | Floxuridine | 79 | 14 |  |  |
| CPD001563707 | Mitoxantrone | 8 | 5 | 31 | 4 |
| CPD001906784 | Enalapril maleate | 100 | 30 |  |  |
| CPD000058337 | Budesonide | 23 | 5 |  |  |
| CPD000466386 | Ramipril | 77 | 25 |  |  |
| CPD000718757 | Depo medrol | 63 | 29 |  |  |
| CPD000058383 | Norepinephrine | 92 | 22 |  |  |
| CPD001491664 | Amcinonide | 57 | 19 |  |  |
| CPD001317855 | Clomid | 13 | 12 |  |  |
| CPD001819784 | Phentolamine | 53 | 9 |  |  |
| CPD000058874 | Fludarabine | 90 | 37 |  |  |
| CPD000109709 | Testosterone | 133 | 61 |  |  |
| CPD000471891 | Isotretinoin | 38 | 10 |  |  |
| CPD000058376 | Methimazole | 58 | 15 |  |  |
| CPD000596519 | Zonisamide | 67 | 15 |  |  |
| CPD000058355 | Brimondidine | 187 | 350 |  |  |
| CPD000036734 | Mebendazole | 52 | 16 |  |  |
| CPD000058736 | Meclizine | 29 | 13 |  |  |
| CPD000146393 | Dilantin | 55 | 22 |  |  |
| CPD000059182 | Miochol | 75 | 14 |  |  |
| CPD000326766 | Dantrolene sodium | 54 | 15 |  |  |
| CPD001227192 | Dexamethasone | 68 | 24 |  |  |
| CPD000394012 | Benztropine mesylate | 5 | 3 | 40 | 15 |
| CPD000058324 | Ganciclovir | 71 | 28 |  |  |
| CPD000059219 | Mesna | 94 | 25 |  |  |
| CPD000058785 | Meclomen | 53 | 9 |  |  |
| CPD000471882 | Fluconazole | 82 | 11 |  |  |
| CPD001453712 | Metaproterenol | 88 | 15 |  |  |
| CPD000071170 | Methoxsalen | 83 | 36 |  |  |
| CPD000058224 | Chloram phenicol | 96 | 32 |  |  |
| CPD000499584 | Tizanididne | 75 | 16 |  |  |
| CPD001453706 | Paroxetine HCl | 6 | 8 | 10 | 7 |
| CPD000550486 | Mirtazapine | 42 | 28 |  |  |
| CPD000010931 | Etomidate | 71 | 20 |  |  |
| CPD000499578 | Moban | 49 | 20 |  |  |
| CPD001453708 | Fluvastatin | 43 | 17 |  |  |
| CPD000058680 | Urecholine | 109 | 20 |  |  |
| CPD001496804 | Cefuroxime | 76 | 19 |  |  |
| CPD000718805 | Cytoxan | 125 | 13 |  |  |
| CPD000550478 | Eszopiclone | 209 | 365 |  |  |
| CPD000058802 | Bendrofluazide | 43 | 19 |  |  |
| CPD000058508 | Raloxifen | 13 | 4 |  |  |
| CPD000058351 | Zidovudine | 49 | 13 |  |  |
| CPD000058365 | Clozapine | 6 | 5 | 56 | 15 |
| CPD001317850 | Amicillin | 62 | 7 |  |  |
| CPD000058800 | Acebutol | 59 | 20 |  |  |
| CPD000058707 | Amoxicilline | 46 | 17 |  |  |
| CPD000857209 | Epinephrine | 50 | 16 |  |  |
| CPD000857239 | Azacytidine | 51 | 10 |  |  |
| CPD000058186 | Buspar | 122 | 250 |  |  |
| CPD000436311 | Rimantadine | 29 | 10 |  |  |
| CPD000059121 | Podofilox | 60 | 14 |  |  |
| CPD000058313 | D-cycloserine | 92 | 30 |  |  |
| CPD000059124 | Cortisone | 50 | 10 |  |  |
| CPD000058295 | Clomipramine | 5 | 4 | 22 | 6 |
| CPD001227191 | Carbamazepine | 76 | 15 |  |  |
| CPD000875213 | Memantine | 35 | 10 |  |  |
| CPD000036827 | Desipramine | 6 | 4 | 28 | 7 |
| CPD000326711 | Mexiletine | 62 | 19 |  |  |
| CPD000058438 | Disopyramide | 65 | 14 |  |  |
| CPD000673569 | Stavudine | 63 | 10 |  |  |
| CPD000097306 | Doxazosin | 21 | 14 |  |  |
| CPD000058963 | Minoxidil | 73 | 6 |  |  |
| CPD000059167 | Propranolol | 10 | 6 | 68 | 6 |
| CPD001496943 | Ribavirin | 38 | 7 |  |  |
| CPD000058309 | Terazosin | 72 | 25 |  |  |
| CPD000058635 | Chlorthalidone | 54 | 38 |  |  |
| CPD000058330 | Methylprednisolone | 46 | 21 |  |  |
| CPD001496977 | Phenelzine | 67 | 13 |  |  |
| CPD000058767 | Naltrexone | 51 | 31 |  |  |
| CPD000469282 | Glycopyrrolate | 42 | 3 |  |  |
| CPD000046147 | Ethambutol | 109 | 28 |  |  |
| CPD001453715 | Cetirizine | 66 | 30 |  |  |
| CPD000539527 | Dicloxacillin | 67 | 13 |  |  |
| CPD000718800 | Meloxicam | 125 | 15 |  |  |
| CPD001906781 | Daunorubicin | 8 | 5 | 32 | 10 |
| CPD001906779 | Rifapentine | 37 | 15 |  |  |
| CPD000274084 | Penicillin | 73 | 31 |  |  |
| CPD000043336 | Gatifloxacin | 94 | 38 |  |  |
| CPD000550475 | Clopidogrel | 56 | 20 |  |  |
| CPD001551784 | Cefotaxime | 109 | 71 |  |  |
| CPD000466319 | Lamivudine | 113 | 45 |  |  |
| CPD001307702 | Ondansetron | 66 | 12 |  |  |
| CPD000339803 | Betamethasone | 83 | 30 |  |  |
| CPD000550473 | Celecoxib | 95 | 54 |  |  |
| CPD000058778 | Aminomethylbenzenesulf | 90 | 19 |  |  |
| CPD001906782 | Thiothixene | 5 | 6 | 75 | 18 |
| CPD000465669 | Citalopram | 44 | 15 |  |  |
| CPD000471864 | Azithromycin | 63 | 24 |  |  |
| CPD000673570 | Lovastatin | 85 | 28 |  |  |
| CPD000326785 | Aminoglutetimide | 129 | 59 |  |  |
| CPD000058452 | Fluoxetine | 1 | 1 | 17 | 9 |
| CPD001233272 | Flunisolide | 73 | 18 |  |  |
| CPD000058225 | Acyclovir | 61 | 21 |  |  |
| CPD000058443 | Etodolac | 48 | 8 |  |  |
| CPD000718785 | Simvastatin | 70 | 13 |  |  |
| CPD001227203 | Rifabutin | 16 | 8 |  |  |
| CPD001496951 | Felodipine | 15 | 7 |  |  |
| CPD000499582 | Quinapril | 69 | 13 |  |  |
| CPD000499573 | Acitretin | 18 | 3 |  |  |
| CPD000718798 | fexofenadine | 64 | 17 |  |  |
| CPD001563899 | Fluoromethadolone | 89 | 18 |  |  |
| CPD000466298 | Sertraline | 1 | 2 | 17 | 5 |
| CPD001566944 | Carbidopa | 81 | 55 |  |  |
