# Supplementary material for: Evaluation of combination therapy for Burkholderia cenocepacia lung infection in different in vitro and in vivo models
Source: PLoS One. 2017 Mar 1;12(3):e0172723. doi: 10.1371/journal.pone.0172723 (PMC5332113; doi:10.1371/journal.pone.0172723)
Supplement: S2 Table — Hits are classified according to their therapeutic indication. Compounds were tested at a concentration of 100 μM in the presence or absence of tobramycin (512 μg/ml). The values shown in the left column represent the mean residual metabolic activity of the compound in the presence of tobramycin compared to treatment with tobramycin alone, and the standard deviation. The values in the right column represent the mean residual metabolic activity of the compound compared to untreated control, and the standard deviation. X means that the compounds did not cause a significant difference in fluorescence signal compared to the signal of untreated biofilms. SNRI: serotonin-norepinephrine reuptake inhibitor, SSRI: serotonin reuptake inhibitor, TCA: tricyclic antidepressant. (DOCX) [file pone.0172723.s002.docx]

**Table S2. Overview of the hits identified after screening the NIH Clinical Collection 1&2 against biofilms of *B. cenocepacia*. Hits are classified according to their therapeutic indication. Compounds were tested at a concentration of 100 μM in the presence or absence of tobramycin (512 μg/ml). The values shown in the left column represent the mean residual metabolic activity of the compound in the presence of tobramycin compared to treatment with tobramycin alone, and the standard deviation. The values in the right column represent the mean residual metabolic activity of the compound compared to untreated control, and the standard deviation. X means that the compounds did not cause a significant difference in fluorescence signal compared to the signal of untreated biofilms. SNRI: serotonin-norepinephrine reuptake inhibitor, SSRI: serotonin reuptake inhibitor, TCA: tricyclic antidepressant**

| # | Pubchem code | Compound | Description | mean | SD | mean | SD |  |
| --- | --- | --- | --- | --- | --- | --- | --- | --- |
|  |  | Anti-infective agents |  |  |  |  |  |  |
| 1 | CPD000058356 | Hexachlorophene | Desinfectant | 0 | 1 | 5 | 6 |  |
| 2 | CPD000471847 | Triclosan | Desinfectant | 3 | 3 | 1 | 1 |  |
| 3 | CPD000058733 | Miconazole nitrate | Antifungal imidazole | 1 | 1 | 48 | 11 | x |
| 4 | CPD001370749 | Econazole nitrate | Antifungal imidazole | 1 | 1 | 21 | 5 |  |
| 5 | CPD000469293 | Oxiconazole nitrate | Antifungal imidazole | 4 | 4 | 65 | 16 | x |
| 6 | CPD000058460 | Ketoconazole | Antifungal imidazole | 6 | 6 | 73 | 9 | x |
| 7 | CPD000466351 | Efavirenz | Antiviral drug | 2 | 2 | 20 | 8 |  |
| 8 | CPD000466367 | Nitazoxanide | Anti-protozoal drug | 6 | 4 | 16 | 13 |  |
| 9 | CPD000875314 | Primaquine phosphate | Anti-protozoal drug | 7 | 5 | 29 | 2 |  |
| 10 | CPD000875233 | Mefloquine HCl | Anti-protozoal drug | 4 | 4 | 11 | 8 |  |
| 11 | CPD000312779 | Chloroxine | Anti-microbial | 5 | 6 | 44 | 11 |  |
|  |  | Antipsychotics and antidepressants |  |  |  |  |  |  |
| 12 | CPD000058411 | Fluphenazine HCl | Phenothiazine | 5 | 4 | 26 | 8 |  |
| 13 | CPD000058180 | Perphenazine | Phenothiazine | 3 | 3 | 46 | 8 | x |
| 14 | CPD000058380 | Thioridazine HCl | Phenothiazine | 0 | 1 | 18 | 3 |  |
| 15 | CPD000059133 | Trifluoperazine HCl | Phenothiazine | 3 | 4 | 20 | 7 |  |
| 16 | CPD000058254 | Chlorpromazine HCl | Phenothiazine | 2 | 4 | 22 | 10 |  |
| 17 | CPD000058855 | Droperidol | Butyrophenone | 9 | 7 | 62 | 10 | x |
| 18 | CPD000449283 | Haloperidol HCl | Butyrophenone | 6 | 1 | 20 | 3 |  |
| 19 | CPD001906782 | Thiothixene | Thioxanthene | 5 | 6 | 75 | 18 | x |
| 20 | CPD000058470 | Loxapine succinate | Dibenzoxazepine | 2 | 5 | 43 | 6 |  |
| 21 | CPD000058365 | Clozapine | Dibenzoxazepine | 6 | 5 | 56 | 15 | x |
| 22 | CPD000466362 | Perospirone HCl | Azopirones | 7 | 4 | 49 | 10 | x |
| 23 | CPD000449282 | Duloxetine | SNRI | 4 | 6 | 20 | 2 |  |
|  | CPD000469136 | Duloxetine HCL | SNRI | 6 | 2 | 10 | 4 |  |
| 24 | CPD000466298 | Sertraline HCl | SSRI | 3 | 5 | 10 | 9 |  |
| 25 | CPD000469181 | N-methylparoxetine | SSRI | 2 | 2 | 31 | 20 |  |
|  | CPD001453706 | Paroxetine HCl | SSRI | 6 | 8 | 10 | 7 |  |
|  | CPD000466269 | Paroxetine maleate | SSRI | 5 | 6 | 23 | 4 |  |
| 26 | CPD000058452 | Fluoxetine HCl | TCA | 1 | 1 | 17 | 9 |  |
| 27 | CPD000058295 | Clomipramine HCl | TCA | 5 | 4 | 22 | 6 |  |
| 28 | CPD000058388 | Imipramine HCl | TCA | 4 | 6 | 36 | 7 |  |
| 29 | CPD000036827 | Desipramine HCl | TCA | 6 | 4 | 28 | 7 |  |
| 30 | CPD000058486 | Nortriptyline HCl | TCA | 2 | 3 | 21 | 3 |  |
| 31 | CPD000058368 | Amitriptyline HCl | TCA | 0 | 0 | 22 | 4 |  |
| 32 | CPD000058416 | Amoxapine | TCA | 5 | 6 | 34 | 5 |  |
| 33 | CPD000058230 | Doxepin HCl | TCA | 2 | 1 | 39 | 7 |  |
| 34 | CPD000449297 | Nefazodone HCl | other | 10 | 14 | 47 | 22 |  |
|  |  | Anticancer therapy and/or hormonal therapy |  |  |  |  |  |  |
| 35 | CPD001906781 | Daunorubicin | Anthracycline | 8 | 5 | 32 | 10 |  |
| 36 | CPD000469210 | Vinorelbine | Antimitotic | 5 | 3 | 22 | 9 |  |
| 37 | CPD001563707 | Mitoxantrone | Type II topoisomerase inhibitor | 8 | 5 | 31 | 4 |  |
| 38 | CPD001491671 | Tamoxifen | SERM | 4 | 9 | 69 | 10 | x |
| 39 | CPD000238204 | Ethylestrenol | Anabol steroid | 6 | 7 | 46 | 9 | x |
| 40 | CPD000058187 | Flutamide | Antiandrogene | 6 | 2 | 36 | 6 |  |
|  |  | Miscellaneous group |  |  |  |  |  |  |
| 41 | CPD000469188 | Montelukast sodium | Leukotriene receptor modulator | 8 | 5 | 85 | 21 | x |
| 42 | CPD000058379 | Promethazine HCl | Histamine antagonist | 4 | 8 | 30 | 10 |  |
| 43 | CPD000469183 | Azelastine HCl | Histamine antagonist | 6 | 7 | 36 | 3 |  |
| 44 | CPD000449274 | AM404 | Metabolite paracetamol | 7 | 9 | 99 | 10 | x |
| 45 | CPD000466272 | Pizotifen meleate | Serotonine antagonist | 7 | 5 | 51 | 9 | x |
| 46 | CPD000387107 | Honokiol | Neolignal biphenol | 5 | 4 | 8 | 3 |  |
| 47 | CPD000469177 | Atomoxetine HCl | SNRI | 5 | 6 | 8 | 3 | x |
| 48 | CPD000469294 | Benproperine | Cough agent | 2 | 4 | 25 | 8 |  |
| 49 | CPD000058366 | Nitrendipine | Ca channel blocker | 8 | 4 | 73 | 10 | x |
| 50 | CPD000440694 | Pterostilbene | Stilbenoid | 5 | 9 | 30 | 10 |  |
| 51 | CPD000471618 | Tegaserod maleate | Irritable bowel syndrome | 4 | 5 | 10 | 4 |  |
| 52 | CPD000449269 | Bifemelane HCl | Choligeric drug | 6 | 2 | 23 | 9 |  |
| 53 | CPD000449292 | Donepezil HCl | Acetylcholinesterase inhibitor | 5 | 4 | 57 | 5 | x |
| 54 | CPD000058466 | Loperamide HCl | Opioid receptor agonist | 2 | 4 | 36 | 7 |  |
| 55 | CPD000058490 | Oxybutynin chloride | Anticholinergic | 8 | 9 | 44 | 9 |  |
| 56 | CPD000058821 | Procyclidine HCl | Anticholinergic | 6 | 7 | 48 | 20 | x |
| 57 | CPD000394012 | Benztropine mesylate | Anticholinergic, benzatropine | 5 | 3 | 40 | 15 |  |
| 58 | CPD000469196 | Tolterodine tartrate | Antimuscarinic | 8 | 6 | 62 | 8 | x |
| 59 | CPD000466346 | Naftodipil | α1-adrenergic R antagonist | 5 | 6 | 24 | 5 |  |
| 60 | CPD000059167 | Propranolol HCl | Beta blocker | 10 | 6 | 68 | 6 | x |
