# Supplementary figures and images for: Evaluation of combination therapy for Burkholderia cenocepacia lung infection in different in vitro and in vivo models
Source: PLoS One. 2017 Mar 1;12(3):e0172723. doi: 10.1371/journal.pone.0172723 (PMC5332113; doi:10.1371/journal.pone.0172723)

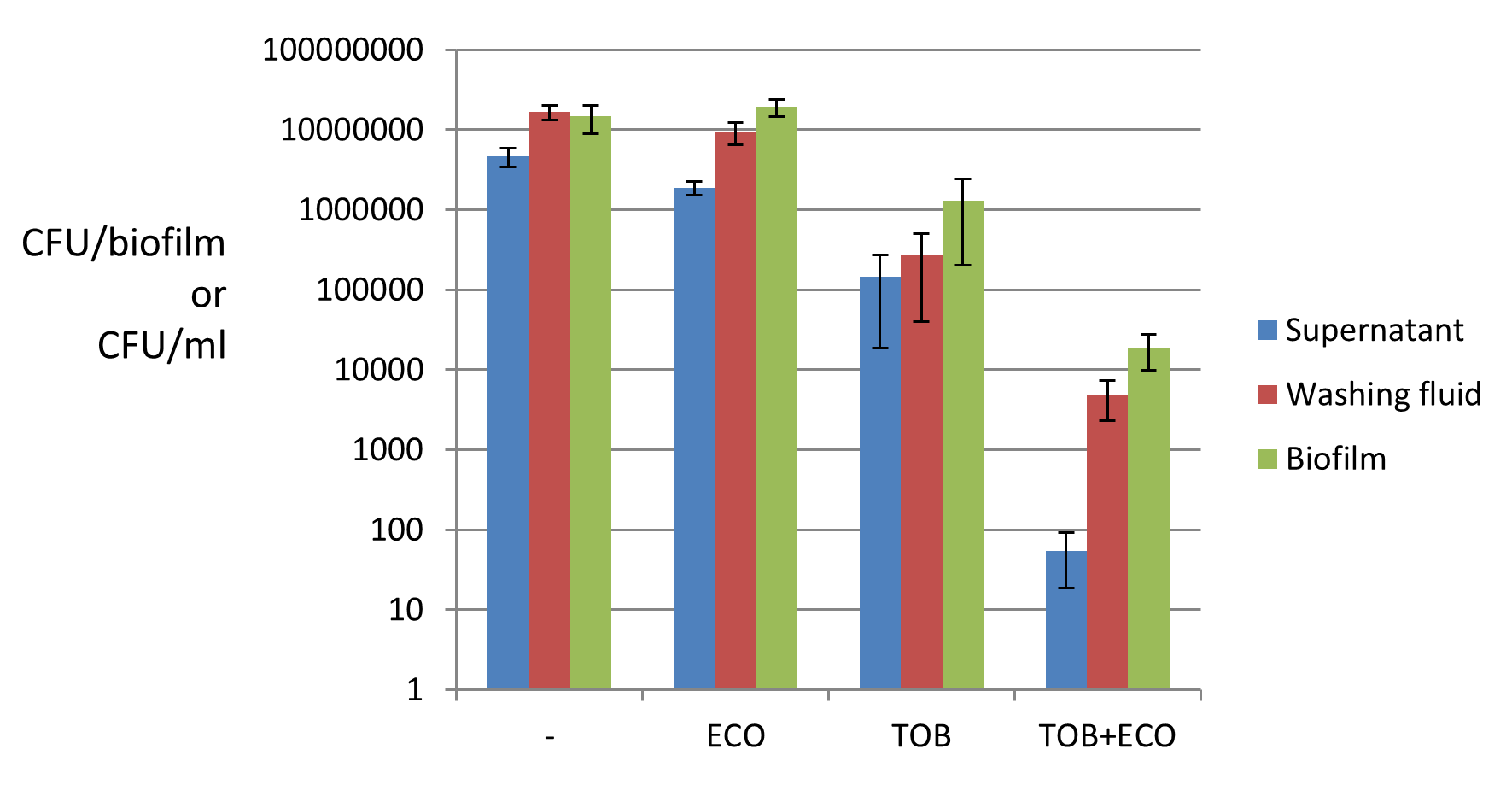

Supplement: S1 Fig — Data shown are log CFU/biofilm or log CFU/ml (for supernatant or washing fluid). The data shown are the average of four biological replicates (error bars represent standard error). (TIF) [file pone.0172723.s003.tif]

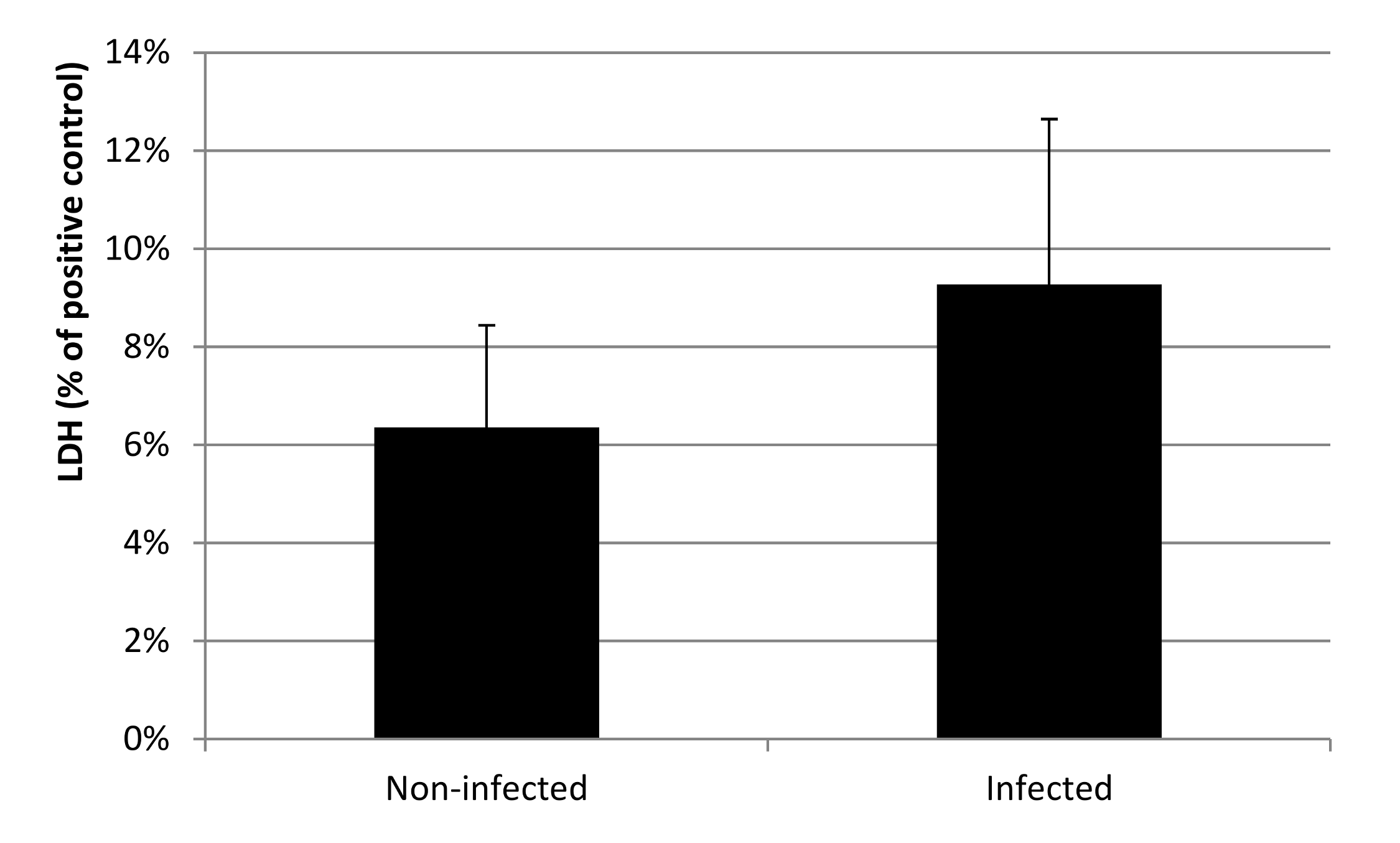

Supplement: S2 Fig — LDH release is presented as a percentage of a positive control (3D lung epithelial cells lysed with Triton-X100). Data shown are average, error bars indicate SD. (TIFF) [file pone.0172723.s004.tiff]

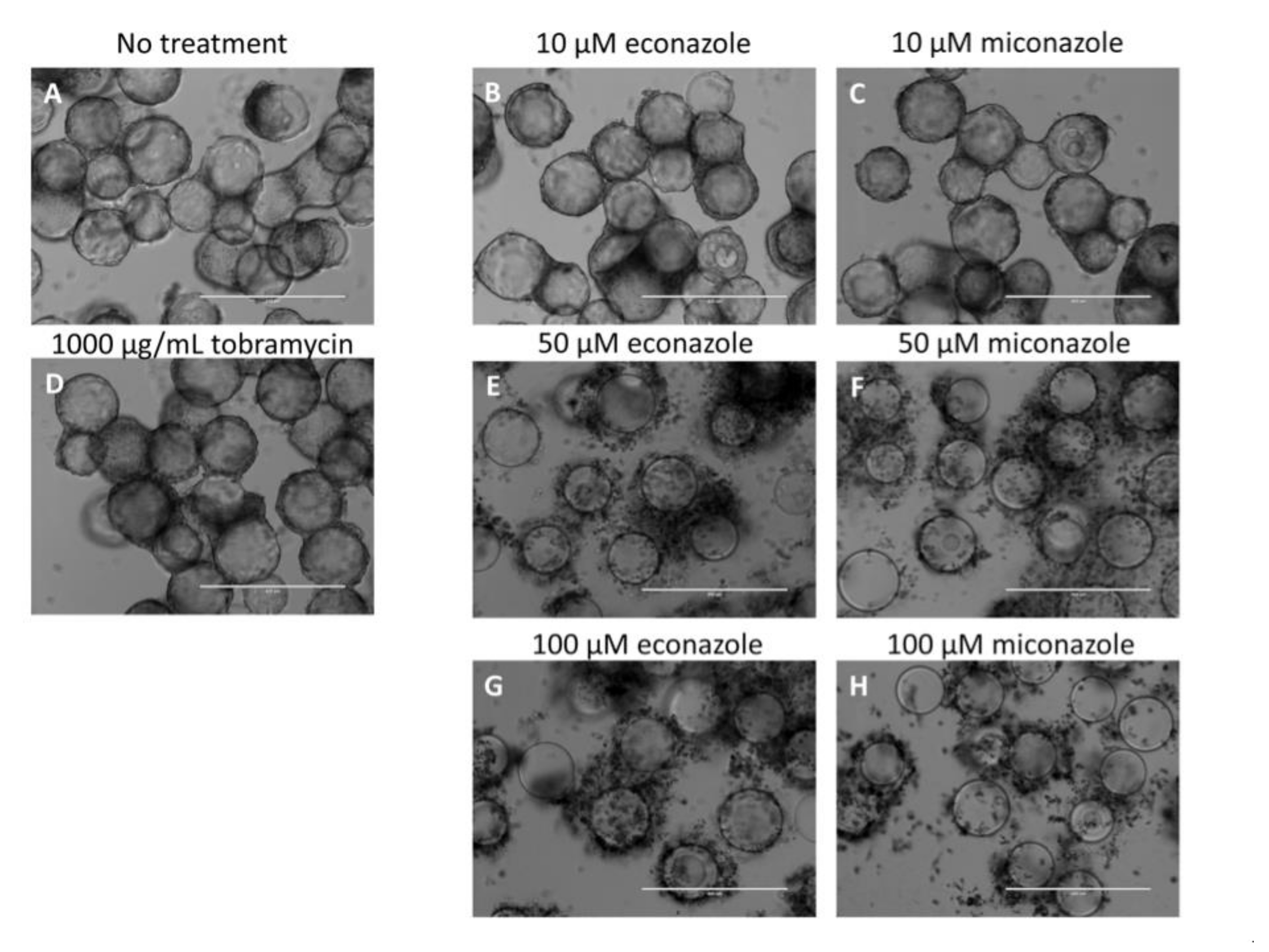

Supplement: S3 Fig — Exposure of 3D A549 lung epithelial cells for 17 h to 10, 50, 100 μM econazole (B, E, G); 10, 50, 100 μM miconazole (C, F, H) or 1000 μg/mL tobramycin (D). A control condition where no antimicrobial agents were added is also included (A). High concentrations of the imidazoles resulted in detachment of host cells from microcarrier beads. Magnification is 300x. Scale bar is 400 μm. (TIFF) [file pone.0172723.s005.tiff]

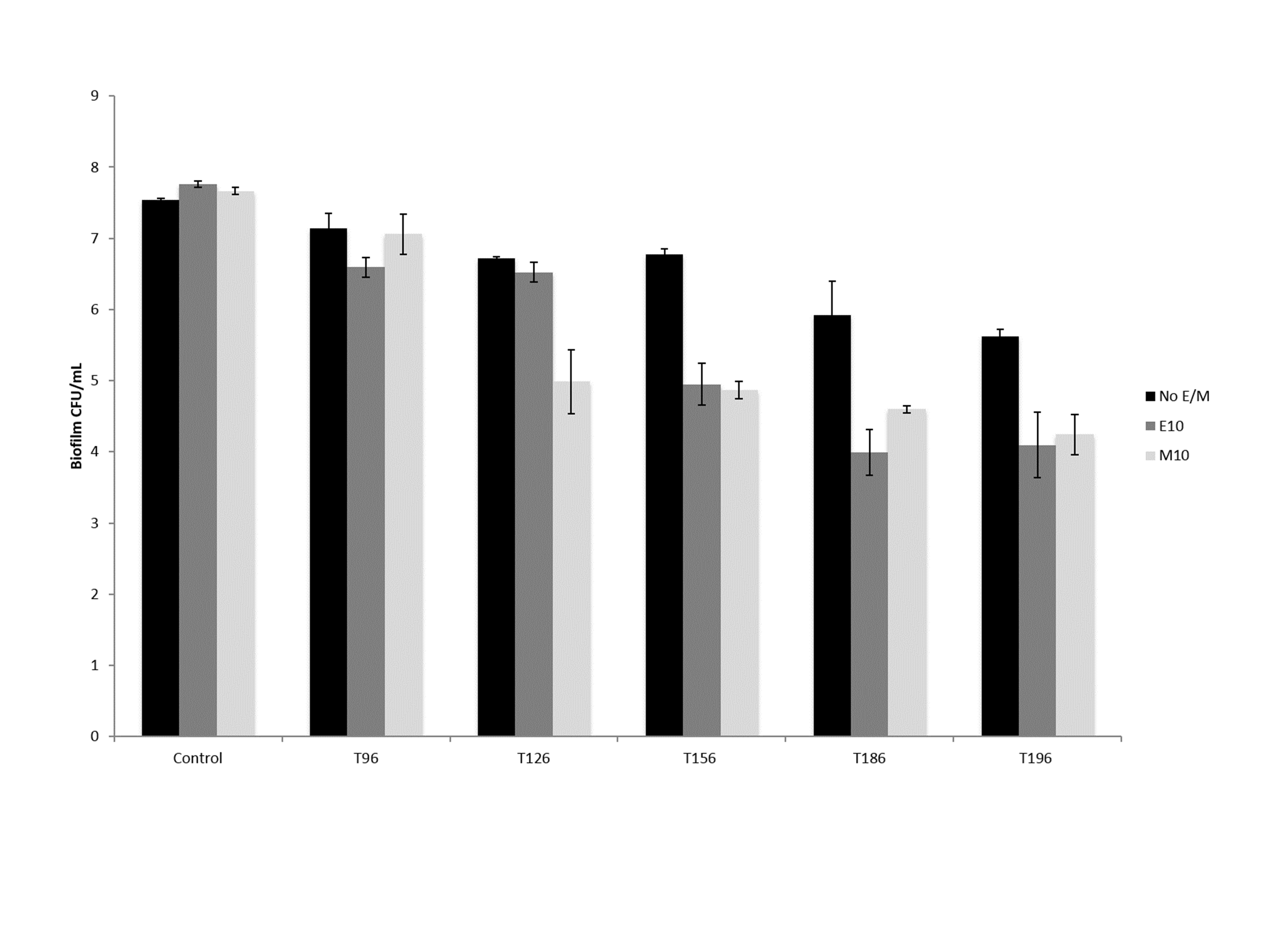

Supplement: S4 Fig — Biofilms of B. cenocepacia K56-2 were grown in 48-well MTP in the presence of tobramycin (T, in μg/ml) with 10μM econazole (E) or miconazole (M) to determine the optimal concentration for the tests in 3D human lung epithelial cells. (Data shown are average, n≥3, error bars indicate SD) (TIF) [file pone.0172723.s006.tif]

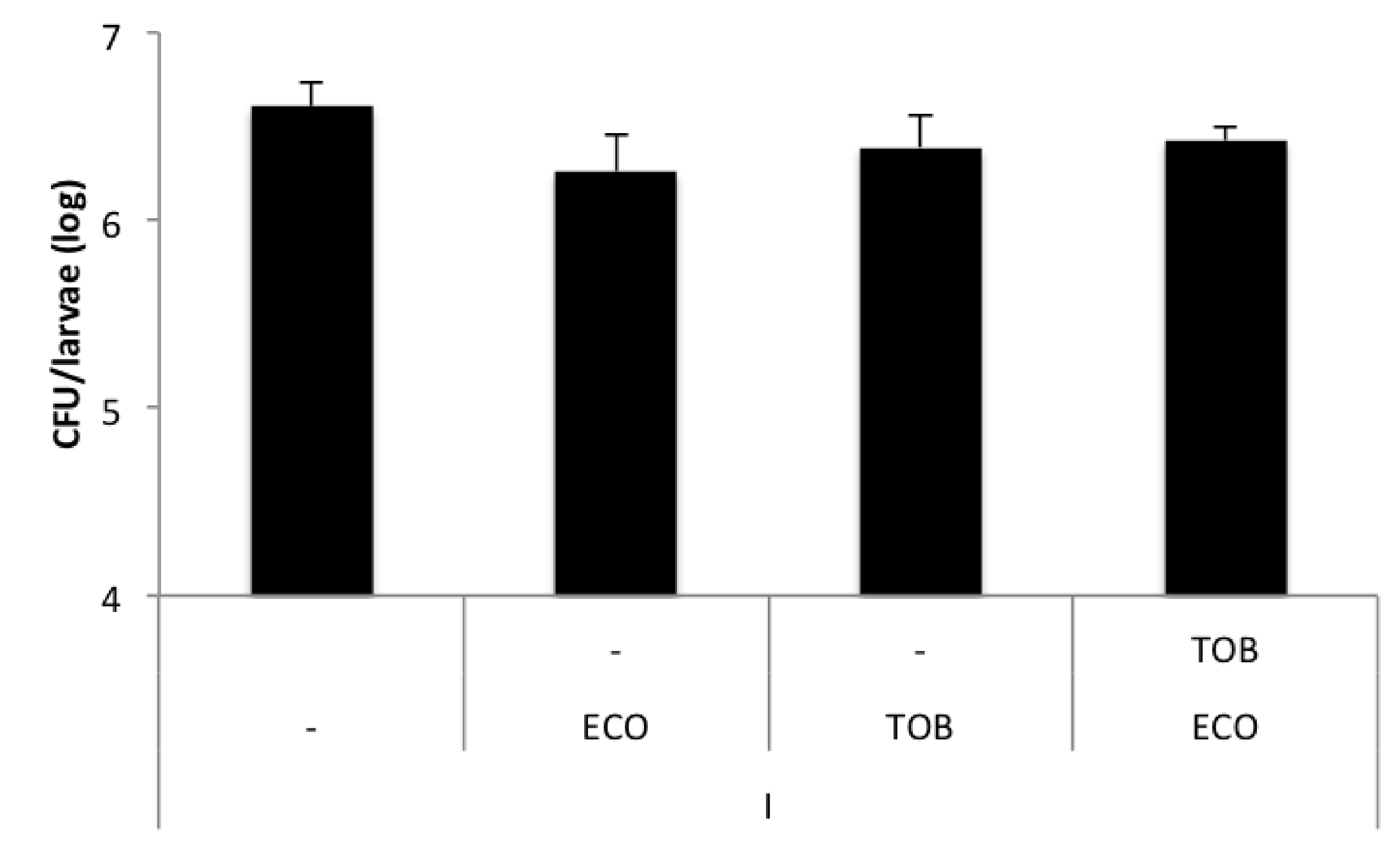

Supplement: S5 Fig — G. mellonella was infected with B. cenocepacia LMG 16656 and treated with tobramycin (TOB), econazole (ECO), or the combination. CFU/larvae was determined 24 h p.i. and treatment. Larvae were homogenized and plated on selective Burkholderia medium. (Data shown are average, n≥3, error bars indicate SEM). (TIFF) [file pone.0172723.s007.tiff]

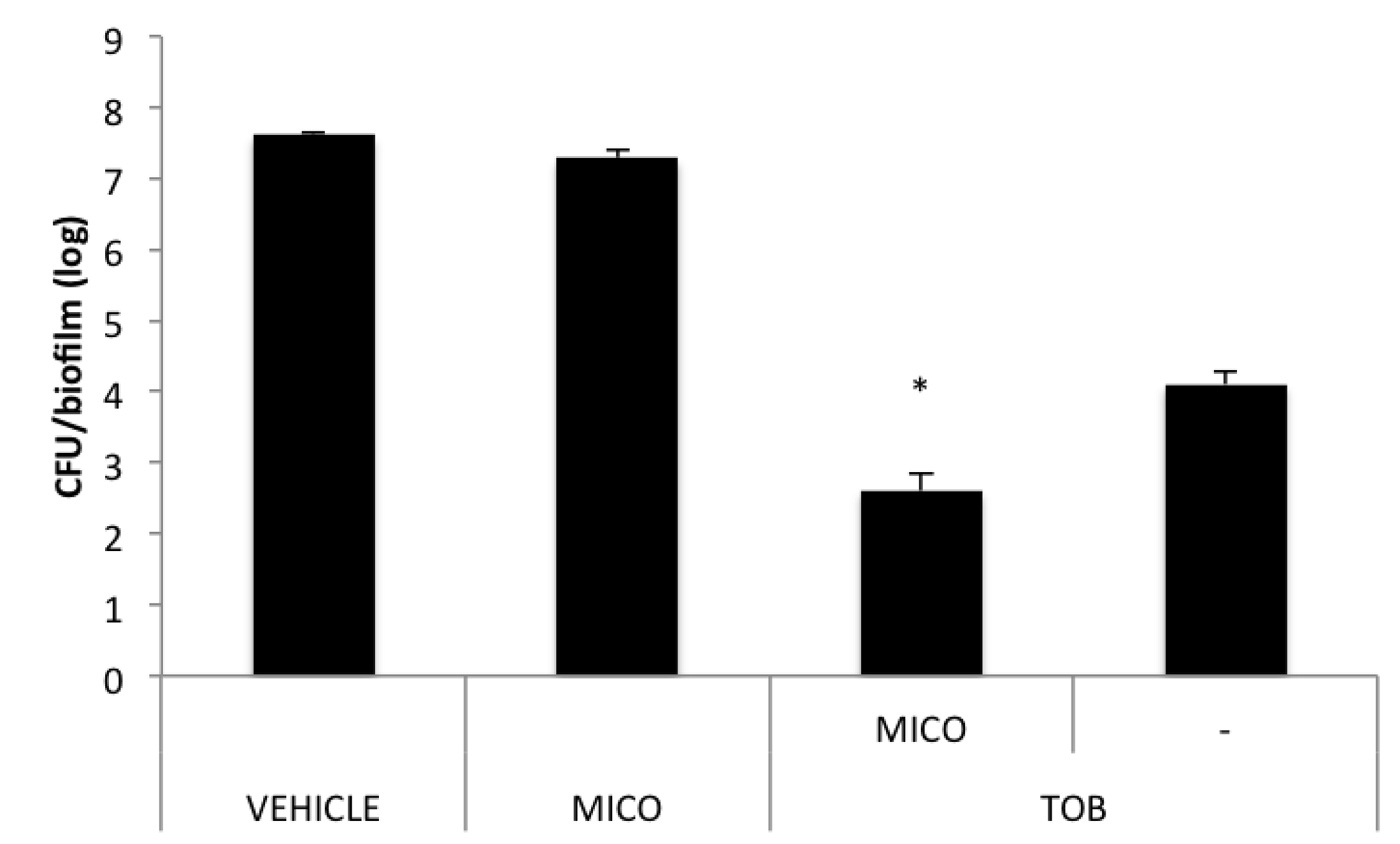

Supplement: S6 Fig — In order to evaluate the antimicrobial activity of a formulation for inhalation prior to use in the mouse lung infection model, biofilms of B. cenocepacia LMG 16656 were formed in 96-well MTPs and subsequently treated with the diluted (129x) formulation. This diluted formulation corresponds to vehicle (tween 80 dissolved in PS), 512 μg/mL tobramycin (TOB), 200 μM miconazole (MICO) or the combination of tobramycin and miconazole. The asterisk indicates a significantly different number in log CFU/BF compared to treatment with tobramycin alone (P value < 0.05). (Data shown are average, n≥3, error bars indicate SEM). (TIFF) [file pone.0172723.s008.tiff]
